# Collagen-based medical devices: technological basis and clinical studies

**DOI:** 10.3389/fbioe.2026.1971640

**Published:** 2026-10-29

**Authors:** Sergei Voloshin, Aliia Giliazova, Egor Rudakov, Elena Istranova, Peter Timashev, Artem Antoshin

**Affiliations:** 1 Institute for Regenerative Medicine, Sechenov First Moscow State Medical University (Sechenov University), Moscow, Russia; 2 Department of Biological Chemistry, Sechenov First Moscow State Medical University (Sechenov University), Moscow, Russia

**Keywords:** applications, clinical studies, collagen, commercial products, implants, technologies

## Abstract

Despite the widespread use of collagen-based medical devices in clinical practice, their clinical efficacy is often analyzed independently from the technological aspects that largely determine the functional capabilities and limitations of a specific product. In this review, the authors formulated technical requirements for collagen-based medical devices across seven clinical areas: dentistry, dermatology, ophthalmology, gastroenterology, neurosurgery, general surgery, traumatology and orthopedics. For each area, clinical studies registered in ClinicalTrials.gov and reported in the relevant scientific literature were analyzed. Product manufacturing technologies and characteristics were identified based on patent searches in the Espacenet and PatentScope databases, as well as data provided by manufacturers. It was demonstrated that collagen-based medical devices should not be regarded as a universal class with an unambiguous regenerative effect. For several clinical indications and specific products, the authors identified neutral or negative clinical outcomes, which were examined from a technological perspective. This approach makes this review valuable for researchers focused on developing new collagen-based products by providing an opportunity to gain both clinical and technical insights and identify relevant research gaps.

## Graphical Abstract

**Figure:**
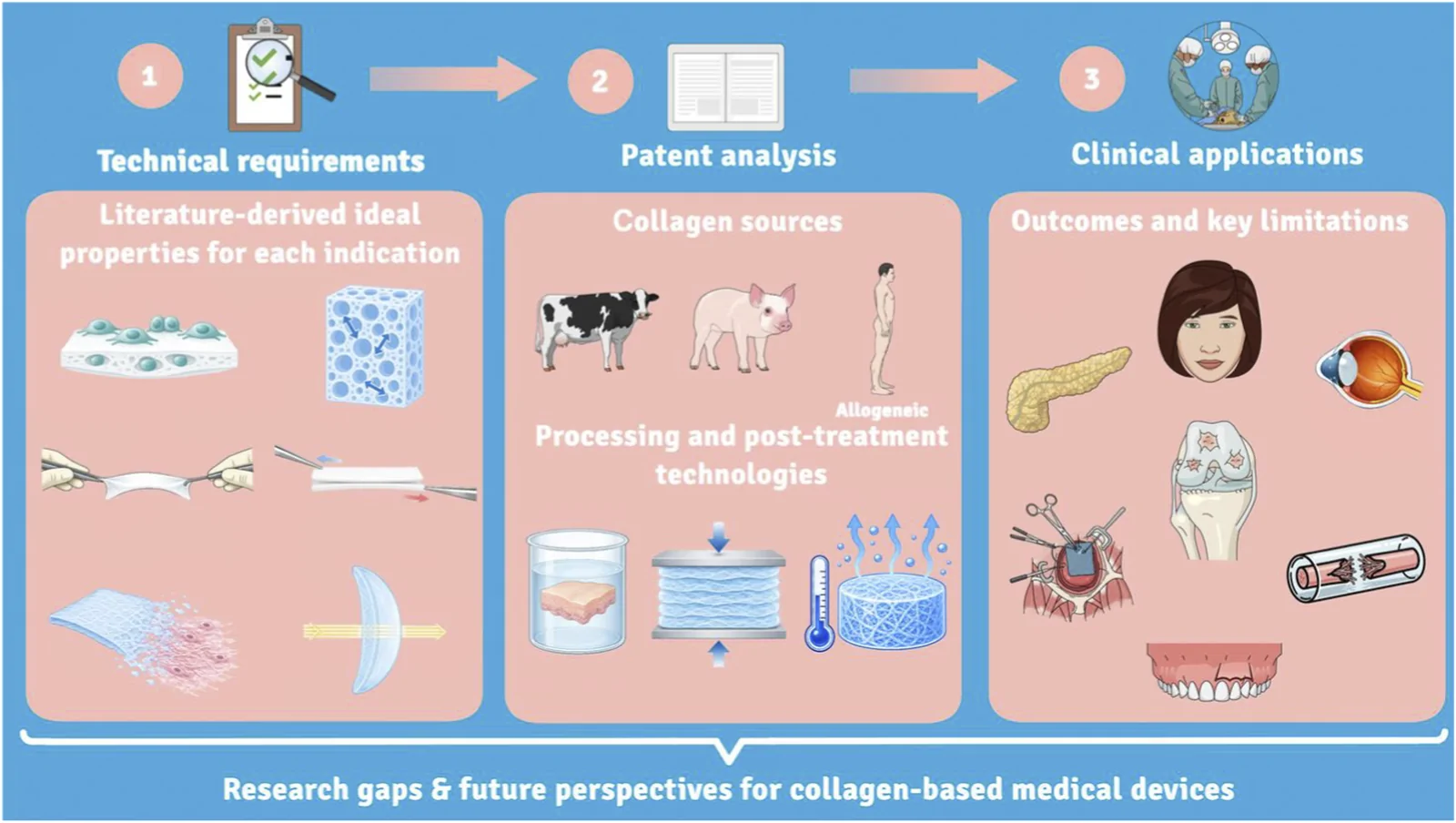

## 1 Introduction

Over the next decade, the global medical device market is predicted to show substantial growth from $540 billion in 2024 to $890 billion by 2030 (Fortune Business Insights, 2026), which is a compelling incentive for researchers to engage in the development of new medical devices. Among different biomaterials applied for this purpose, collagen is the most extensively studied and widely utilized due to its excellent biocompatibility, biodegradability, and pro-regenerative potential (Shekhter et al., 2019). It is usually derived from animal sources and can be subsequently processed into gels, membranes, powders, and sponges, making it highly versatile for various medical applications (Silvipriya et al., 2015).

Thousands of collagen-based products have been commercialized across multiple fields, including dentistry, dermatology, cosmetology, traumatology, orthopedics, ophthalmology, cardiovascular surgery, and urology (Wang et al., 2023). Nevertheless, further development of collagen medical devices is still ongoing: new clinical areas are explored and new production technologies are investigated, for example, relying on the principles of synthetic biology (Liu et al., 2022).

Although a great number of reviews on clinical trials of collagen-based medical devices exist in the literature, most of them have no defined methodology for article retrieval, and this process is rather arbitrary and author-specific (Rezvani Ghomi et al., 2021; Zhu et al., 2022), which may result in incomplete coverage of all possible clinical applications of collagen medical devices (Abedi et al., 2024). Moreover, the reviews are usually narrowly focused on the specific organ, e.g., skin (Akira Tashiro et al., 2020), bone (Fan et al., 2023; Abtahi et al., 2023), nerve tissue (Roccuzzo et al., 2021), gum (Naomi et al., 2020), as well as on collagen devices produced from the specific animal source (Gallo et al., 2020). Finally, the reviews usually pay significant attention to clinical experience, but often lack or insufficiently highlight technological aspects: the manufacturing principles, the specific technical characteristics, and the required “ideal” parameters for their high clinical efficiency (Wang et al., 2023).

Therefore, in this review, we focused not only on the results of clinical trials, but also on the technological aspects of collagen-based medical devices. Specifically, the manufacturing technologies were described based on relevant patent analysis, as well as the desired device parameters were determined for each clinical nosology. Finally, it is generally reported that collagen is a “universal remedy” that improves regeneration of all types of tissues and organs, and it has practically no disadvantages (Chen et al., 2023). Here, we critically evaluated clinical trial results and demonstrated that not all collagen-based products are the “panacea”, and in these cases, the future device improvements are required based on the deep analysis of the core technologies.

Thus, this review integrates clinical and technological evidence to identify product-specific limitations and research gaps. Alternatively, this work may also help to build the research strategy for those who are developing the medical devices from other biocompatible and clinically approved biomaterials based on the revealed disadvantages of collagen products.

## 2 Study design and methods

The discussion of products started from the brief overview of their desired “ideal” technical parameters or mechanisms of action for each nosology. Further, the detailed information on product collagen source and its key processing methods was given. For clinical study analysis, collagen-based medical devices were divided into three groups according to their form: membrane, sponge, or injectable (Figure 1). The main clinical applications and functions of representative products analyzed in this review are summarized in Table 1.

**FIGURE 1:**
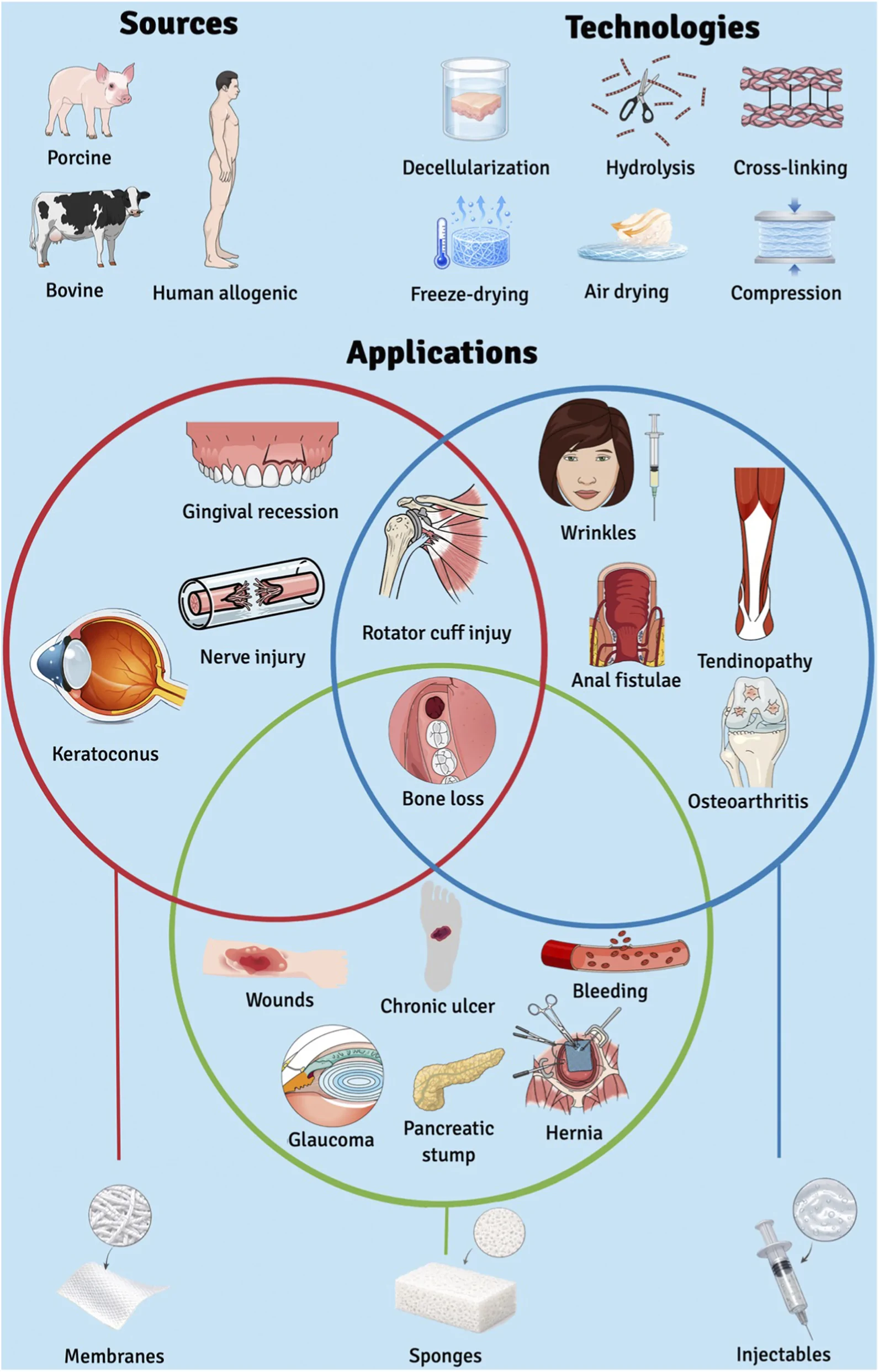
Overview of collagen extraction sources, manufacturing technologies, and clinical applications related to each product form.

**TABLE 1:** Clinical applications and key functional contributions of different forms of collagen-based medical devices.

Product form	Clinical application	Representative products	Key function
Membrane	Peripheral nerve repair	NeuraGen®, NeuroMatrix®, Neuroflex®, AxoGuard®, Remplir™	Guidance and protection of regenerating peripheral nerves; bridging or wrapping of nerve defects (Arslantunali et al., 2014; Chan et al., 2025)
	Guided bone regeneration	Bio-Gide®, Striate+®, Jason membrane®, Creos Xenoprotect®, EZ Cure®, Vitala®	Barrier between bone defect and soft tissue; prevention of soft-tissue ingrowth while supporting bone regeneration (Tai et al., 2023)
	Periodontal/peri-implant soft-tissue regeneration	Mucograft®, Ossix Volumax®, PriMatrix®	Matrix for soft-tissue ingrowth and maintenance of regenerated tissue volume (Tavelli et al., 2000)
	Ophthalmic applications	LinkCor®, XENIA®, ABCcolla®; Visian®	Restoration of corneal curvature, its thickness and visual function; optical correction as intraocular lenses (Bhattacharjee et al., 2023; Borja et al., 2008; Hackett et al., 2011; Fagerholm et al., 2014; Rafat et al., 2023; Mirazul Islam et al., 2015)
	Rotator cuff repair	REGENETEN®, CelGro®, GraftJacket®, ArthroFLEX®, Conexa®	Tendon augmentation; bioinductive support and/or mechanical reinforcement (Hitchens et al., 2026)
Sponge	Dental bone regeneration	Bio-Oss® Collagen, Ossix Bone®, Osteogen Plug®, NuOss XC®	Porous osteoconductive matrix supporting bone regeneration (Keil et al., 2021; Canullo et al., 2023)
	Ophthalmic surgery	Ologen®	Maintenance of the filtration space and fluid flow while reducing postoperative scar formation (Yuan et al., 2015)
	Wound healing	Integra®, OASIS® Wound Matrix, PuraPly®, PROMOGRAN®, NeoMatriX®	Matrix for cell infiltration, promotion of granulation tissue formation and subsequent wound closure (Davis et al., 2022)
	Prevention of pancreatic fistula	TachoSil®, HEMOPATCH®	Sealing and hemostasis of pancreatic resection surfaces (Park et al., 2016)
	Abdominal-wall reinforcement	Strattice®	Reinforcement of abdominal-wall defects and prevention of hernia recurrence (Cheng et al., 2014)
	Dural repair	Lyoplant®, HEMOPATCH®	Dural defect coverage and prevention of cerebrospinal fluid leakage (Nowak et al., 2019)
	Surgical hemostasis and sealing	TachoSil®, HEMOPATCH®, Lyostypt®	Blood absorption, hemostasis, and tissue sealing (Di Carlo et al., 2011)
	ACL repair	BEAR® Implant	Retention of endogenous growth factors and provision of a regenerative bridge between torn ACL ends (Shah et al., 2025)
	Bone regeneration/spinal fusion	INFUSE® (InductOs®), HEALOS®	Delivery of osteoinductive or osteoconductive components supporting bone formation (Burkus et al., 2002)
	Cartilage repair	NOVOCART® 3D	Provides a three-dimensional porous collagen scaffold for autologous chondrocyte delivery and cartilage regeneration (Lukas et al., 2014)
Injectable	Dermal augmentation	Collost®, Zyplast®, Cosmoplast®, Evolence®, BellaFill®, FULLSGEN®	Restoration of soft-tissue volume and stimulation of extracellular-matrix regeneration (Salvatore et al., 2023)
	Dental bone regeneration	OsteoBiol® MP3, OsteoBiol® Gel 40	Ready-to-use delivery of collagen and bone granules into bone defects (Tecnoss Dental Srl, n,d,)
	Anal fistula treatment	Permacol®	Fills and stabilizes the fistula tract to promote tissue healing and fistula closure (Brunner et al., 2019)
​	Tendinopathies	MD-Tissue Collagen®, MD-Shoulder Collagen®, RegenSeal®	Local collagen delivery for supporting tendon extracellular-matrix remodeling and improving pain and functional outcomes (Jong-Ho et al., 2020)
	Cartilage repair	CartiFill®, CartiZol®, MD-Tissue Collagen®, MD-Knee®, Fibroquel®, BioCartilage®	Supports cartilage defect filling and cartilage extracellular-matrix formation while improving pain and joint function (Kim et al., 2021; Cole et al., 2021)

Information on the technical requirements and production technologies of collagen medical devices was obtained from publicly available sources: data from manufacturers and relevant patents obtained from the Espacenet and PatentScope databases. The patent numbers related to each product are given in Supplementary Materials.

A structured literature search was performed to identify clinical studies on collagen-based medical devices. Clinical trials were retrieved from the ClinicalTrials.gov database using the search query “collagen AND device”. The search was conducted without restrictions on publication date.

To identify corresponding peer-reviewed publications, trial identifiers (NCT numbers) were used to conduct search in Scopus, PubMed, and Google Scholar databases. In case no publications were linked to a specific clinical trial, additional searches were performed using product name and keywords related to the device and clinical indication. Studies were excluded if they were preclinical or only *in vitro*.

To improve the completeness of trial retrieval, the search was performed iteratively. Product and manufacturer names identified from the initial broad query, as well as from the patent search described above, were used to conduct a second, targeted round of search directly in ClinicalTrials.gov to reveal additional collagen-based devices whose trial records were indexed primarily by proprietary product or manufacturer names and did not contain the terms “collagen” or “device”. Trials identified through either round were included if collagen was confirmed as the core structural material of the tested device.

## 3 Manufacturing strategies for collagen-based medical devices

### 3.1 Manufacturing strategies and their functional implications

Collagen-based medical devices are usually manufactured by a sequence of operations acting at three different hierarchical levels. At the molecular level, the extraction conditions define the state of the collagen molecule and its nativity. At the tissue level, the native extracellular matrix can be preserved by decellularization or disintegrated into the suspension followed by collagen reconstitution into a membrane, a porous body or a hydrogel. At the compositional level, collagen may be additionally stabilized by cross-linking and/or supplemented with other components. The main manufacturing approaches, their effects on final device properties, and representative commercial products are summarized in Table 2. Most products combine up to three technological operations, while several products comprise only one step (e.g., Avance®, ABCcolla®, GraftJacket®, ArthroFLEX®, Conexa™, OASIS®, Collost®, RegenSeal®, CartiFill®, CartiZol® etc.).

**TABLE 2:** Main manufacturing approaches and their effects on the properties and functions of collagen-based medical devices.

Manufacturing approach		Effect on device properties	Functional contribution	Representative products		
				Membranes	Sponges	Injectables
Decellularization of collagen-rich tissues		Preservation of native collagen-rich ECM architecture and tissue-derived structural organization (Hussey et al., 2018; Crapo et al., 2011)	Supports native-like cell–matrix interactions and tissue regeneration while retaining the structural function and biological activity of the original ECM (Hussey et al., 2018; Crapo et al., 2011)	AxoGuard® Nerve Protector/Connector; Avance®; Creos™ Xenoprotect; Bio-Gide®; Vitala®; Jason membrane®; DynaMatrix®; PriMatrix®; ABCcolla®; XENIA®; CuffPatch™; Restore®; GraftJacket®; ArthroFLEX®; Conexa™; TissueMend®; Zimmer® Collagen Repair Patch; OrthADAPT®; Striate+™/CelGro™; Remplir™	NeoMatriX®; PuraPly™ AM; OASIS®; Strattice™; Lyoplant®	Permacol® collagen paste
Collagen extraction	Enzymatic	Removal of telopeptides and production of purified atelocollagen with reduced native intermolecular cross-links (Meyer, 2019)	Reduces telopeptide-associated antigenicity while enabling reconstitution into application-specific collagen matrices (Meyer, 2019)	NeuraGen® Nerve Guide; NeuraGen® 3D Nerve Guide Matrix	Ossix Bone®; Ologen™; Integra® Dermal Regeneration Template; Helicoll®	Zyplast®; Evolence®; RegenSeal®; CartiFill®; CartiZol®; Fibroquel®
	Alkali treatment			NeuraGen® Nerve Guide; NeuraGen® 3D Nerve Guide Matrix; NeuroMatrix®; NeuroFlex®; Visian® ICL	Integra® Dermal Regeneration Template	Collost®
	Acidic	Greater preservation of the native collagen molecular structure, including telopeptide regions, with subsequent purification by salt precipitation (Darvish, 2022; Babloyan et al., 1985)	Preserves collagen self-assembly capacity, enabling formation of fibrillar matrices with native-like organization (Darvish, 2022; Babloyan et al., 1985)	NeuroMatrix®; NeuroFlex®	​	​
Foaming		Formation of a highly porous, sponge-like collagen structure through incorporation of gas bubbles into the collagen matrix (Shi et al., 2026)	Generates interconnected pores and high surface area, facilitating fluid uptake and interaction with cells and biological fluids (Shi et al., 2026)	​	TachoSil®	​
Air/vacuum drying		Formation of a dense, low-porosity collagen structure through dehydration and compaction of the collagen matrix (Vernon et al., 2005)	Provides thin, mechanically stable structures with barrier and tissue-support functions (Vernon et al., 2005)	Remplir™; Creos™ Xenoprotect; Mucograft®; Vitala®; PriMatrix®; Striate+™; LinkCor®; TissueMend®; CelGro™	TachoSil®; NeoMatriX®; Strattice™	​
Lyophilization	Multi-directional	Formation of a highly porous, non-aligned structure with controllable porosity and high fluid absorption capacity (O’Brien et al., 2004)	Facilitates fluid uptake, cell infiltration, and three-dimensional tissue ingrowth (O’Brien et al., 2004)	NeuraGen® Nerve Guide; NeuroMatrix®; NeuroFlex®; OSSIX® Volumax; Mucograft®; Vitala®; Jason membrane®; DynaMatrix®; PriMatrix®; REGENETEN®; TissueMend®;	Bio-Oss® Collagen; OSSIX® Bone; Hemocollagene®; NuOss XC®; Ologen™; Integra® Dermal Regeneration Template; NeoMatriX®; PROMOGRAN®; HEMOPATCH®; Lyoplant®; HEALOS®;	​
	One-directional	Formation of an anisotropic structure with aligned collagen pores or fiber bundles (O’Brien et al., 2004; Allan et al., 2021)	Guides directional cell growth and tissue regeneration (O’Brien et al., 2004; Allan et al., 2021)	NeuraGen® 3D Nerve Guide Matrix	NOVOCART® 3D	​
Mechanical stretching				Remplir™, Striate+™, CelGro™	​	​
Pressing		Compaction of the collagen matrix, resulting in increased density, reduced porosity, and improved mechanical strength (Wilson et al., 2022; Abou et al., 2006)	Provides thin, mechanically stable structures and allows adjustment of implant geometry and structural properties (Wilson et al., 2022; Abou et al., 2006)	OSSIX® Volumax; XENIA®	NuOss XC®; Helicoll®	​
		Increased and controllable implant thickness and mechanical stability through combination of multiple layers (Kumar et al., 2013)	Provides long-term volume and structural stability during regeneration of larger soft-tissue defects (Kumar et al., 2013)	AxoGuard® Nerve Protector/Connector; DynaMatrix®		
Multilayer construction				AxoGuard®; OSSIX® Volumax; Mucograft®; DynaMatrix®; REGENETEN®; CuffPatch™; Restore®;	NOVOCART® 3D	​
Cross-linking	Glutaraldehyde	Formation of additional intermolecular cross-links and strengthening of the collagen network (Lin et al., 2020; Adamiak and Sionkowska, 2020; Tirella et al., 2012; Tai et al., 2024)	Provides prolonged structural persistence, but may increase the risk of cytotoxicity and adverse host response associated with residual cross-linker (Adamiak and Sionkowska, 2020)	​	Integra® Dermal Regeneration Template	Zyplast®, Cosmoplast®
	EDC/NHS		Prolongs structural persistence with no cross-linker-associated cytotoxicity (Lin et al., 2020; Adamiak and Sionkowska, 2020; Tirella et al., 2012)	LinkCor®, CuffPatch™, OrthADAPT®	PuraPly™ AM	​
	GLYMATRIX®			Ossix Volumax®	Ossix Bone®	Evolence®
	UV			Vitala®, LinkCor®, XENIA®	​	​
	Multi-agent		Provides enhanced long-term structural stability and prolonged functional persistence while limiting residual cross-linker-associated cytotoxicity (Tai et al., 2024)	​	​	Sunmax FULLSGEN®
	Other or non-specified cross-linking agents		Increases mechanical stability and resistance to enzymatic degradation, thereby prolonging structural persistence (Lin et al., 2020; Adamiak and Sionkowska, 2020; Tirella et al., 2012; Tai et al., 2024)	NeuraGen® Nerve Guide; NeuraGen® 3D Nerve Guide Matrix; NeuroMatrix®; NeuroFlex®; Zimmer® Collagen Repair Patch	Ologen™	Fibroquel®
Incorporation of additional components		Addition of application-specific biological, physicochemical, or mechanical properties (Widrig et al., 2007; Harris et al., 2017; Cullen et al., 2002; G et al., 2023; Spotnitz, 2010; Pieper et al., 2002; Ryan and O’Brien, 2015; Yannas et al., 1989; Murad et al., 1981; Karnina et al., 2021; Wahl and Czernuszka, 2006)	Provides application-specific functions, including regenerative stimulation, hemostasis, osteogenic activity, or mechanical reinforcement (Widrig et al., 2007; Harris et al., 2017; Cullen et al., 2002; G et al., 2023; Spotnitz, 2010; Pieper et al., 2002; Ryan and O’Brien, 2015; Yannas et al., 1989; Murad et al., 1981; Karnina et al., 2021; Wahl and Czernuszka, 2006)	NeuraGen® 3D Nerve Guide Matrix, Creos™ Xenoprotect, Visian® ICL	Bio-Oss® Collagen, OSSIX® Bone, NuOss XC®, OsteoGen Plug®, Ologen™, Integra® Dermal Regeneration Template, PuraPly™ AM, PROMOGRAN®, HEMOPATCH®, TachoSil®, HEALOS®	Zyplast®, CosmoPlast®, Sunmax FULLSGEN®, BellaFill®, OsteoBiol® Gel 40, OsteoBiol® MP3, MD-Tissue®, MD-Shoulder®, MD-Knee®, Fibroquel®

At the molecular level, the chemical extraction process influences both collagen antigenicity and its ability to self-assemble into fibrils. Acid extraction preserves the telopeptide regions and the amide groups of asparagine and glutamine, allowing collagen molecules to reassemble into fibrils with a D-periodic organization similar to that of the native tissue. At the same time, the potentially antigenic determinants located in the telopeptides are also preserved (Davison et al., 1967; Pontz et al., 1970). Enzymatic treatment cleaves the telopeptides and yields atelocollagen with reduced antigenicity. However, this process also removes regions involved in native intermolecular cross-linking and subsequent ordered formation of fibrils (Holmes et al., 2017; Shayegan et al., 2016; Kuznetsova and Leikin, 1999). Alkaline treatment is typically performed in the presence of a saturated salt solution, and it removes telopeptides together with the deamidation of amine residues. It results in collagen, which is soluble at physiological pH but unable to form native banded fibrils (Hattori et al., 1999; Darvish, 2022).

Consequently, the latter two collagen extraction options are common for injectables that are represented by atelocollagen-based (Zyplast®, Evolence®, CartiFill®, Fibroquel®) and alkali-treated products (Collost®). For them, the host response is the main safety concern, while the required mechanical function is minimal and the loss of fibril-forming capacity is not critical. For membranes, sponges and conduits, in contrast, all extraction approaches are possible, and devices of the same type may be produced from acid-extracted (NeuroMatrix®, NeuroFlex®) or enzymatically extracted/alkali-treated collagen (NeuraGen®, NeuraGen® 3D Nerve Guide Matrix). In these products, the deficit of mechanical characteristics resulting from the molecular level is further compensated by cross-linking step.

At the tissue level, decellularization remains the dominating production method for membranes, while sponges and injectables are mainly produced from extracted and reconstituted collagen. Obviously, the membrane form is simple and can be obtained from native tissues (like skin, pericardium, SIS) with preserved ECM architecture and biological activity (Palmosi et al., 2022). If greater thicknesses or volume is required, the membranes are assembled into multilayer constructs, comprising membrane-membrane interfaces (DynaMatrix®) or a dense membrane combined with a porous sponge layer (Mucograft®). For the alignment of collagen fibers in membranes, the mechanical stretching is used by one company (Orthocell) for several of its products (Remplir™, Striate+™, CelGro™) to enhance their cell-guidance characteristics (Christophe et al., 2015).

The preparation of highly porous three-dimensional structures by lyophilization allows the pore size to be controlled within the range of tens to hundreds μm via freezing conditions (Haugh et al., 2010). As well, in this process, a sufficiently sharp unidirectional freezing gradient transforms the isotropic structure into an anisotropic one with aligned channels (Pot et al., 2015). However, aligned-channel architecture is rare across the products, and is represented by only two of them (NeuraGen® 3D Nerve Guide Matrix and NOVOCART® 3D).

At the compositional level, cross-linking is used to stabilize the collagen network, and Table 2 shows a clear generational shift in its chemistry depending on the product entry on the market. Glutaraldehyde was used in the earliest generation (1985–2003) of devices (Zyplast®, Cosmoplast®, Integra® Dermal Regeneration Template). It reacts with the ε-amino groups of lysine and hydroxylysine residues and introduces exogenous five-carbon bridges that remain in the material, while unreacted monomer and aldehyde polymers can subsequently be released from it (Speer et al., 1980). Products introduced from 2000 and later adopted zero-length and “biological” cross-linking. EDC/NHS is a zero-length cross-linker that activates the carboxyl groups of glutamate and aspartate residues to form direct amide bonds with lysine, and it is fully washed from the material (LinkCor®, CuffPatch™, OrthADAPT®, PuraPly™ AM) (Olde Damink et al., 1996). UV irradiation (Vitala®, XENIA®) is another alternative forming cross-links through photo-oxidation of aromatic side chains, mainly dityrosine bonds between adjacent tyrosine residues (Fan et al., 2024). The sugar-based GLYMATRIX® technology (Ossix® family) reproduces the non-enzymatic glycation that cross-links collagen as in ageing tissue using D-ribose, and during material degradation only biocompatible glycated protein fragments are left (Lin et al., 2020). Most recently (2019 and later), multi-agent strategies (Sunmax FULLSGEN®) combining bridging- and zero-length cross-links were applied: сombining submaximal doses of each reagent allows for comparable implant biostability with a lower residual reagent load than that achieved with maximum cross-linking using a single reagent (Islam et al., 2021).

Incorporation of additional components is a versatile approach to broaden the functionality of the collagen device, for example, its regenerative stimulation, hemostatic and osteogenic activity, and mechanical stability. Moreover, in some products (Bio-Oss® Collagen, NuOss XC®, OsteoBiol® MP3), their functional position reverses: collagen plays a supporting role providing cohesion and handling, while mineral-containing bone substitutes provide the main biological activity. However, this “reassignment” of collagen role has direct clinical consequences, since the resorption of these two components is not synchronized.

### 3.2 Manufacturing-dependent host response and biodegradation

Despite the generally low immunogenicity of collagen (Liu et al., 2019a), the method used to obtain the collagen matrix can influence its immunogenicity and inflammatory response. In case of decellularization, the host response may result from residual cellular and antigenic components rather than collagen itself. Incomplete removal of cellular material, DNA, and xenogeneic epitopes such as α-Gal may contribute to inflammatory responses to decellularized extracellular matrices (Parenteau-Bareil et al., 2010). Quantitative acceptance criteria have been proposed for cell removal: residual dsDNA below 50 ng per mg of matrix dry weight, DNA fragments shorter than 200 bp, and no visible nuclear material in stained sections (Crapo et al., 2011). However, these criteria do not cover the matrix-bound xenogeneic epitopes: α-Gal remains associated with the collagen matrix after decellularization and requires a separate enzymatic step with α-galactosidase for its removal, with the achievable degree of removal depending on the enzyme source and on the tissue form being processed (Yu-Yue et al., 2025).

For extracted collagen, the antigenicity of the retained telopeptide regions makes the collagen source the dominant variable of the host response, which is particularly evident for injectable fillers. Bovine collagen fillers have historically been associated with hypersensitivity reactions, with reported incidences of approximately 3%–5%, requiring pretreatment skin testing (SHOSHANI et al., 2007). Porcine collagen was subsequently introduced as an alternative source, and it has demonstrated lower immunogenicity in clinical studies (SHOSHANI et al., 2007; Monstrey et al., 2007).

Cross-linking influences the host response through two independent mechanisms: the density of the resulting polymer network and the reagent residues left in the material. Strongly cross-linked collagen matrices may demonstrate reduced cellular infiltration and remodeling and prolonged macrophage and foreign-body giant-cell presence, potentially resulting in chronic inflammation and fibrous encapsulation (Anderson et al., 2008). Residues of cross-linking agents or byproducts of the treatment process may further contribute to cytotoxicity and trigger inflammatory reactions, while in the case of glutaraldehyde, the implant calcification may be additionally observed (Delgado et al., 2015). Clinically, the combined effect of residual antigenic material and extensive cross-linking has been reported for decellularized matrices used in rotator cuff repair (Permacol®, CuffPatch™), where inflammatory and foreign-body responses restricted cellular infiltration and interfered with constructive remodeling.

The structural integrity of the collagen matrix, the method used to extract and reconstitute collagen, and, more importantly, the degree of implant cross-linking directly affects the rate of its biodegradation. Cross-linking increases resistance to enzymatic degradation and is particularly important when prolonged structural stability is required as exemplified by dermal fillers and barrier membranes (Adamiak and Sionkowska, 2020; Tal et al., 2008; KLIGMAN and ARMSTRONG, 1986). However, there is no universal optimal degradation time, as the device should generally maintain its structural and functional properties for the period required to perform its intended function and subsequently allow for tissue remodeling or replacement (Mndlovu et al., 2024). Consequently, substantially different degradation profiles are required for collagen devices used in different clinical applications (Table 3).

**TABLE 3:** Application-specific degradation requirements for collagen-based medical devices.

Сlinical application	Required degradation profile	Rationale
Peripheral nerve regeneration	Approximately 4–8 months (Gaudin et al., 2016)	Protects and guides the regenerating nerve throughout the relatively slow process of axonal regeneration, followed by gradual replacement of the conduit with native tissue (Arslantunali et al., 2014; Chan et al., 2025)
Guided bone regeneration	Approximately 2–6 months (Mizraji et al., 2000)	Prevents soft-tissue ingrowth into the defect during the main period of bone regeneration (Tai et al., 2023)
Periodontal and peri-implant soft-tissue regeneration	Several months (Herford et al., 2010)	Supports the formation and maturation of new soft tissue while preserving matrix volume during cellular infiltration and remodeling (Tavelli et al., 2000)
Corneal regeneration (keratoconus)	Long-term/years (Rafat et al., 2016; Xeroudaki et al., 2020)	Ensures long-term transparency, thickness stability, and mechanical support of the corneal stroma (Rafat et al., 2016; Xeroudaki et al., 2020)
Glaucoma surgery	Approximately 3–6 months (Marey et al., 2018)	Forms a three-dimensional matrix that preserves the subconjunctival space and facilitates fluid flow during bleb formation and tissue remodeling (Yuan et al., 2015)
Hemostasis and tissue sealing	Short-term (Di Carlo et al., 2011)	Achieves rapid bleeding control and/or tissue sealing during the early postoperative period rather than supporting long-term tissue regeneration (Di Carlo et al., 2011)
Bone regeneration	Degradation synchronized with new bone formation, typically over approximately 3–6 months (Dewey and Harley, 2021)	Stabilizes the defect volume while serving as an osteoconductive matrix for new bone formation (Burkus et al., 2002)
Dermal augmentation	>12 months is desirable (NARINS et al., 2008)	Sustains soft-tissue volumization and contour correction over an extended period (Salvatore et al., 2023)

### 3.3 Post-manufacturing evaluation

The immunogenicity, biodegradation profile, and functional performance of collagen-based medical devices should be established through a combination of *in vitro* characterization and preclinical studies before their clinical application and regulatory approval (Huang et al., 2026; Wang et al., 2026). The scope of biological tests of safety is determined individually for each device type according to: the duration of body contact, material composition, manufacturing process, degradation behavior, and the biological data already available for the material (ISO 10993–1, ISO 14971).

Cytotoxicity can be evaluated using direct-contact, indirect-contact, or extract-based assays according to ISO 10993–5, while preparation of test samples and extracts is addressed by ISO 10993–12. Chemical characterization of the device identifies relevant constituents, manufacturing residues, extractables, and leachables, and it is followed by toxicological risk assessment according to ISO 10993–18, ISO 10993–17. For degradable devices, degradation products should also be evaluated (ISO 10993–9).

Biological evaluation should be complemented by characterization of properties relevant to the intended device function (ASTM F2150 (Wang et al., 2026)). ASTM F2212 provides guidance for characterization of type I collagen used as a starting material for surgical implants and tissue-engineered medical products. Other properties of the collagen medical device commonly include enzymatic degradation (ASTM F3089), mechanical properties (ASTM F3260, ISO 7198), microstructure or porosity (ASTM F2450), fluid absorption, swelling, and water-vapor transmission rate (EN 13726, ASTM E96/E96M, ASTM F2900) (Ghodbane and Dunn, 2016; Solderer et al., 2023; Shi et al., 2020; Verdolino et al., 2026).

Following *in vitro* characterization, the safety, host response, biodegradation, and functional performance of the device are evaluated *in vivo* (Liu et al., 2022). Animal studies should comply with the animal-welfare requirements of ISO 10993–2 and Good Laboratory Practice (GLP) principles (OECD Principles on Good, 2026). Where applicable, national regulatory frameworks should be considered. For example, in the United States, nonclinical safety studies intended to support FDA submissions are subject to requirements of GLP under 21 CFR Part 58. FDA guidance recommends that the selected animal model and its anatomical, physiological, and pathological characteristics should adequately simulate the intended clinical application (General Considerations for Animal Studies, 2026).

Local tissue responses including inflammation, foreign-body reaction, tissue integration, vascularization, and responses to material degradation or absorption can be assessed according to ISO 10993–6. Functional performance, however, should be evaluated in application-specific models. Importantly, there is no universal animal model suitable for all collagen-based medical devices. Rat sciatic nerve-gap models are widely used for initial evaluation of peripheral nerve conduits (Vela et al., 2020), while materials for bone regeneration are usually assessed in critical-size defects (Wei et al., 2024; Li et al., 2015). Studies on rotator-cuff injury often progress from rodent screening to larger animals when clinically relevant surgical and biomechanical performance must be reproduced (Wanlu et al., 2022). Similarly, cartilage-regeneration guidance recommends progression from small-animal screening to large-animal models for later-stage evaluation (Hurtig et al., 2011).

## 4 Membranes

### 4.1 Neurosurgery

Peripheral nerve injuries frequently result in painful neuropathies, since native nerve regeneration process may be incomplete or impaired, which represents a significant clinical challenge in neurology and reconstructive surgery (Daly et al., 2011). Collagen-based nerve conduits are being actively investigated as the instrument for guiding and promoting nerve growth. They serve as tubular bridges between severed nerve ends and limit secondary damage caused by surrounding tissue compression thus protecting nerve regeneration (Yang. et al., 2024a).

An ideal nerve conduit is generally considered to possess adequate mechanical strength (Young’s modulus of 8–16 MPa and tensile strength of 6.5–8.5 MPa), an interconnected porosity of 50%–70%, pore sizes in the range of 5–30 μm, and a wall thickness of 200–600 μm. In addition, the conduit should closely match the diameter of the injured nerve, exhibit minimal swelling, and undergo biodegradation over a period of approximately 4–8 months (Chiono and Tonda-Turo, 2015).

For nerve gaps exceeding 3 cm, the regenerative capacity of nerve conduits becomes substantially limited (Figure 2A). For such gaps, several limiting mechanisms are evident: (1) fibrin cables fail to form or become attenuated as a result of central fibrin matrix contraction, which leads to their characteristic hourglass morphology; (2) the absence of a viable extracellular matrix impairs the formation of Bands of Büngner required to support Schwann cell migration and axonal alignment; (3) the dilutional reduction of the neurotrophic factors’ effective concentration within the conduit lumen leads to hindered transition of biochemical guidance cues (Selim et al., 2021). For overcoming this regenerative barrier, conduits are expected to more closely replicate the structural organization and microenvironment of native nerve tissue (Lu et al., 2021).

**FIGURE 2:**
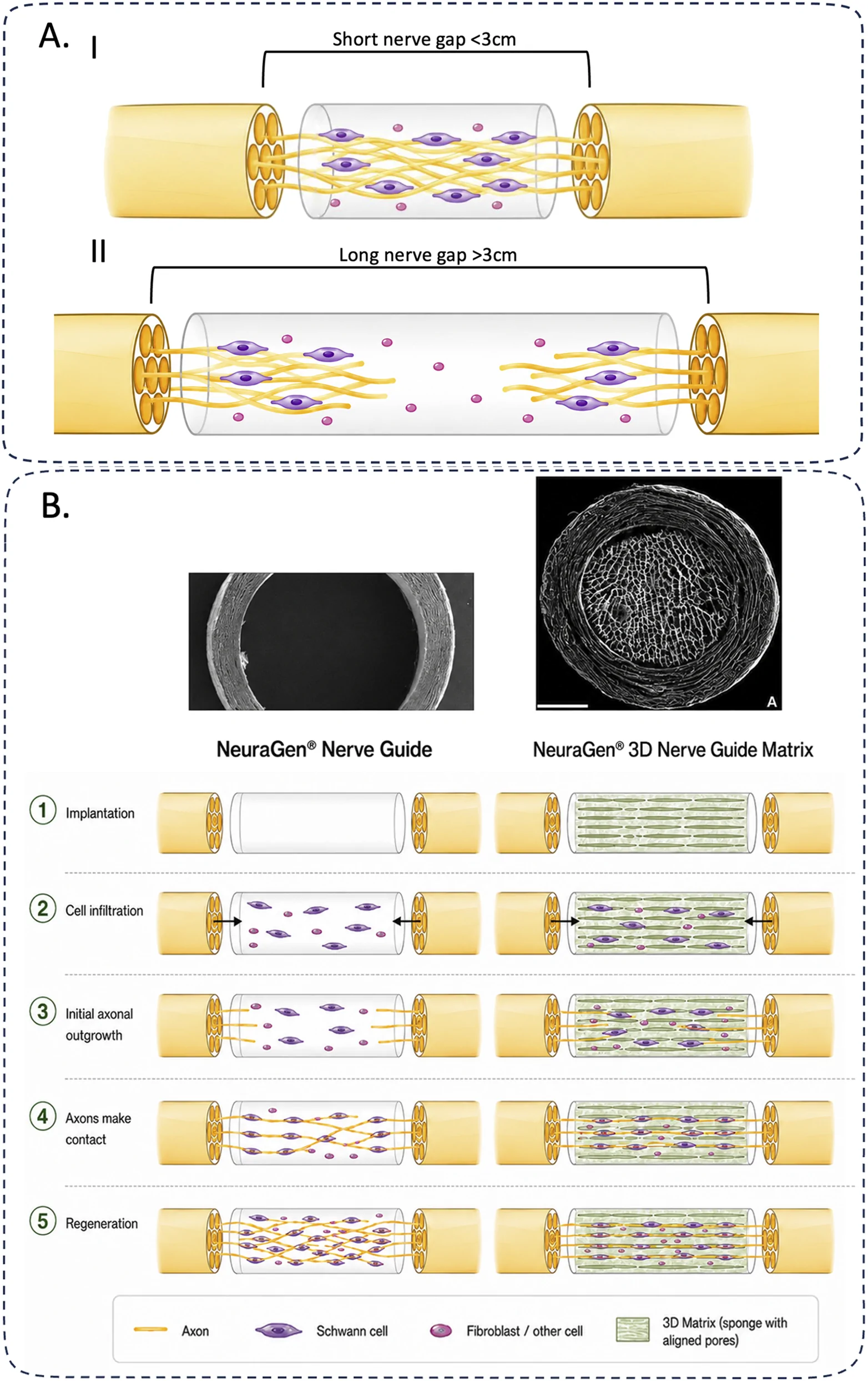
**(A)** Schematic illustration of peripheral nerve regeneration using nerve guidance conduits, comparing short (<3 cm) and long (>3 cm) nerve gaps. **(B)** Microarchitecture and proposed regeneration mechanism of hollow versus aligned matrix nerve guides. Top row: SEM images comparing hollow NeuraGen® Nerve Guide and aligned matrix-containing NeuraGen® 3D Nerve Guide Matrix. Bottom row: proposed mechanism of peripheral nerve regeneration for each device. Reproduced with the permission from (Sagentia, n.d.; NeuraGen® Nerve Guide, 2026).

#### 4.1.1 Product information

Axoguard® Nerve Protector/Nerve Connector are made by the decellularization of porcine small intestinal submucosa (SIS) (Supplementary Table S1a). AxoGuard® Nerve Protector is aimed at peripheral nerve injury with zero gap, and it is suitable for wrapping. AxoGuard® Nerve Connector is a hollow tube applied for peripheral nerve discontinuity with the gap up to 5 mm (Axogen Corporation, 2026a; Axogen Corporation, 2026b). Another example is Avance® Nerve Graft (Axogen) which is a decellularized human nerve allograft with preserved laminin-lined endoneurial tubes that serve as native guidance channels for regenerating axons (U.S. Food and Drug Administration, 2026b).

Remplir® represents a different type of nerve repair device. It is a single-layer, non-cross-linked porcine collagen membrane that is wrapped around the injured nerve. The membrane is produced by decellularization, followed by acid treatment, mechanical stretching to align collagen fiber bundles, and air-drying under tension. This processing results in a thin pliable membrane with distinct rough and smooth surfaces.

Alternatively, collagen membranes may be produced using reconstitution-based methods. Examples include the NeuraGen® Nerve Guide and NeuraGen® 3D Nerve Guide Matrix that are fabricated from bovine collagen extracted by pepsin digestion. Unlike the hollow NeuraGen® Nerve Guide conduit, the NeuraGen® 3D Nerve Guide Matrix contains an internal uniaxially-aligned porous collagen matrix that provides additional structural guidance for growing axons for better functional recovery (Figure 2B) (Sagentia, n.d.).

Other hollow collagen-based conduits are NeuroMatrix® and NeuroFlex®. Both consist of type I bovine collagen extracted by alkali-salt method. Of note, NeuroFlex® differs from NeuroMatrix® by its corrugated wall architecture that provides enhanced flexibility and allows for bending up to approximately 60° without lumen occlusion (Stryker Orthopaedics, 2026).

Lyophilization remains the primary structuring technique used to obtain the final implant design (NeuraGen® Nerve Guide, NeuraGen® 3D Nerve Guide Matrix, NeuroMatrix®, and NeuroFlex®). In contrast, Remplir® retains a membrane architecture through mechanical stretching and subsequent air-drying under tension.

To further enhance functional performance, manufacturers may incorporate additional components into collagen matrices, such as chondroitin-6-sulfate, which is included in the NeuraGen® 3D Nerve Guide Matrix. Chondroitin-6-sulfate is the extracellular matrix molecule upregulated in response to nerve injury, and it has been shown to facilitate Schwann cell migration (Liu et al., 2006).

#### 4.1.2 Clinical performance

The majority of registered clinical trials have not yet reported published outcomes. In these studies, products are compared either with no treatment (NCT03941327, evaluating Axoguard® Nerve Protector for the protection of the spinal accessory nerve) or with alternative conduit-based devices (NCT05339594, comparing NeuraGen® Nerve Guide and NeuraGen® 3D Nerve Guide Matrix for the treatment of peripheral nerve injuries).

A direct comparison of two conduits (AxoGuard® Nerve Connector, and NeuraGen® Nerve Guide) in lingual nerve microsurgery revealed no clinical superiority: postoperative two-point discrimination was 9.67 ± 2.13 mm for AxoGuard® and 9.32 ± 2.96 mm for NeuraGen®, with no significant difference between the groups, while all patients achieved functional sensory recovery (Wilson et al., 2017).

Clinical performance of Remplir™ was evaluated in a prospective case series with a 2-year follow-up involving 19 patients and 36 upper-extremity peripheral nerve reconstructions performed using nerve transfer or nerve grafting. Functional motor recovery of the most proximal target muscles was observed in 76% of cases at 12 months and increased to 85% at 24 months. For all target muscles, functional motor recovery was achieved in 61% and 75% at 12 and 24 months, respectively. However, the study lacked a comparative control group, limiting direct comparison with other nerve repair devices (O’Beirne et al., 2024).

Clinical outcomes of nerve conduits appear to depend on the length of the nerve gap and are generally inferior to processed nerve allografts. In a randomized pilot study comparing processed nerve allograft (Avance® Nerve Graft) with hollow nerve conduits for digital nerve reconstruction, the s2PD at 12 months was 5 ± 1 mm for the allograft and 8 ± 5 mm for the conduit group (NCT00948025 (MeansJr Kenneth et al., 2016)). Similar results were demonstrated in a systematic review, where allografts resulted in s2PD < 15 mm in 100% of repairs compared with 72% for conduits, while the complication rate was 3.0% and 10.9%, respectively (Mauch et al., 2019).

The difference between these approaches becomes more pronounced with increasing nerve gap length. In a multicenter matched cohort study, meaningful sensory recovery was achieved in 88% of processed nerve allograft repairs compared with 61% of conduit repairs. For gaps of 15–25 mm, these values decreased to 85% and 45%, respectively (Leversedge et al., 2020). A systematic review and meta-analysis of 1559 nerve repairs also demonstrated higher meaningful sensory recovery for allografts compared with conduits in short sensory nerve gaps (87.1% vs. 62.2%, respectively) (Lans et al., 2023). However, the available evidence remains limited by the predominance of observational studies and relatively small comparative cohorts, and a systematic critical appraisal classified the certainty of evidence comparing processed nerve allografts with other repair methods as very low (Frostadottir et al., 2023).

### 4.2 Dentistry

Сollagen membranes are widely used for guided bone regeneration (GBR) as well as for the regeneration of soft tissues. In GBR procedures, collagen membranes function as barrier materials by preventing the ingrowth of epithelial and connective tissues into the bone defect site, thereby enabling undisturbed bone regeneration. To ensure this role, the membrane should exhibit sufficient tensile strength to maintain structural integrity during surgical handling and throughout the healing process, typically in the range of 2–8 MPa, while its thickness after hydration generally lies between 0.3 and 1 mm (Kozlovsky et al., 2009; Antoshin et al., 2023a).

Adequate porosity is required to support diffusion of nutrients and metabolic products. Of note, the required pore size depends on the membrane architecture and the function of each membrane surface. Collagen membranes may have a homogeneous porous structure or an asymmetric bilayer architecture (Sbricoli et al., 2020). In bilayer membranes, the side facing the soft tissue has a more compact structure to limit fibroblast and epithelial cell penetration, whereas the porous side facing the bone defect supports cell migration and tissue integration (Allan et al., 2021). Experimental bilayer collagen membranes with pore sizes of 20–50 μm in the compact layer and 200–300 μm in the porous layer have also been developed, demonstrating that substantially different pore sizes can be used depending on the function of each layer (Zhao et al., 2020). The bioresorption period of collagen membranes used in GBR typically ranges from 2 to 6 months, which is generally consistent with the physiological timeline of bone healing (Antoshin et al., 2023a; Sheikh et al., 2017).

In addition to GBR, collagen membranes are employed to increase soft tissue thickness in periodontal and peri-implant applications. Membranes are usually thicker (1–5 mm) and exhibit higher porosity (up to 96%) compared with those used for GBR, reflecting their primary function of supporting soft tissue ingrowth and regeneration (Webb et al., 2022). The membranes are required to maintain structural integrity over several months to ensure long-term volume stability of the newly regenerated soft tissue, which remains one of the key clinical challenges in periodontal and peri-implant therapy (Rothamel et al., 2014; Antoshin et al., 2023b).

#### 4.2.1 Product information

Decellularized-tissue membranes include PriMatrix® (bovine dermis) and several porcine-derived products, such as Bio-Gide® (peritoneum), Vitala® and Jason membrane® (pericardium), DynaMatrix® (SIS), Creos™ Xenoprotect and Striate+™. To achieve the desired implant thickness, multiple decellularized layers may be laminated or cross-linked together, as exemplified by Vitala® (Supplementary Table S1b).

Alternatively, collagen matrices may be manufactured using reconstitution-based methods, which allow for greater control over the final material properties. Representative products include EZ Cure® (porcine type I and III collagen), Ossix Volumax® (porcine collagen), and Mucograft® (porcine type I collagen). These membranes may also exhibit a multilayer architecture, as in Ossix Volumax® and Mucograft®. Lyophilization represents the primary processing technique used to obtain the final membrane structure (Mucograft®, Vitala®, PriMatrix®), while vacuum drying has been reported as the intermediate stage for the production of individual layers in Mucograft®.

Striate+™ (Orthocell) is manufactured using an approach analogous to that employed for Remplir™, the nerve repair device produced by the same company. Accordingly, Striate+® exhibits similar structural characteristics, including an organized collagen architecture with distinct porous and dense surfaces. In Striate+™, the porous surface faces the bone defect to support tissue integration, whereas the dense surface acts as a barrier to soft tissue ingrowth.

To enhance mechanical performance, manufacturers employ cross-linking strategies: the proprietary GLYMATRIX technology (Ossix Volumax®), ultraviolet irradiation (Vitala®), and other cross-linking approaches (EZ Cure®).

#### 4.2.2 Clinical performance

Сollagen membranes as barrier materials for alveolar ridge preservation demonstrate comparable clinical outcomes. Specifically, 4 months after implantation in combination with freeze-dried bone allograft (FDBA), no significant differences were observed between reconstituted and decellularized collagen matrices (Mucograft® and DynaMatrix®, respectively) with respect to vertical bone loss of the buccal plate, which ranged up to 0.7–1.0 mm (NCT01808794 (Parashis et al., 2016)). Comparable results were reported in NCT02697890 (Natto et al., 2017) where Mucograft® or barrier collagen sponge were applied over FDBA graft, and no intergroup differences were reported in terms of buccal vertical reduction.

Similarly, in horizontal bone augmentation, Bio-Gide® applied over Bio-Oss® and Jason membrane® applied over β-tricalcium phosphate (β-TCP) showed no significant differences in vertical or horizontal bone gain, complete defect filling, complications, chair-time, or postoperative pain at 12 months (Merli et al., 2015) and after 3-year follow-up (Merli et al., 2018). Since both the membrane and bone filler varied between the groups, the discussed results are not sufficient to judge the superiority of either membrane. Other studies on GBR as well reported equivalent clinical performance of available commercial membranes (Creos Xenoprotect® vs. Bio-Gide®, NCT02373787 (Wessing et al., 2017)), even when collagen membrane (EZ Cure®) was compared to synthetic poly (lactic-co-glycolic acid) membrane (Carlos et al., 2022).

In vertical ridge augmentation, collagen membranes were used as adjunctive barrier materials over non-resorbable meshes (NCT04257097 (Cucchi et al., 2025; Cucchi et al., 2024)). Reinforced polytetrafluoroethylene (PTFE) mesh with Vitala® and titanium mesh with Bio-Gide® showed similar vertical bone gain (5.79 ± 1.71 mm vs. 5.18 ± 1.61 mm) and healing complication rates (12.5% vs. 8.3%).

In addition to comparative studies of established collagen membranes, the clinical feasibility of newer GBR membranes has been evaluated in prospective studies. Striate+™ was initially assessed in 10 patients receiving 16 dental implants with simultaneous GBR. At 4–6 months, sufficient newly regenerated bone was observed at all 16 implant sites, allowing placement of the healing abutment and subsequent crown placement and loading. No device-related adverse events or wound infections were reported; one case of wound dehiscence occurred but required no intervention and did not affect bone regeneration (ACTRN12615000027516 (Allan et al., 2021)). More recently, the clinical performance of Striate+™ was assessed in a larger post-market study involving 99 patients and 143 dental implants placed in combination with GBR between 2018 and 2023. The study evaluated peri-implant marginal bone levels in relation to patient-, implant-, and procedure-related factors and reported an overall implant success rate of 98.6%, providing further clinical evidence for the use of Striate+® in GBR procedures (Outstanding 98, 2026).

However, collagen membranes appear less effective when act as the main regenerative scaffold rather than as a barrier. When Ossix Volumax® was used solely as a matrix for GBR and compared with FDBA covered by Bio-Gide® for lateral alveolar ridge augmentation, the latter approach demonstrated superior outcomes (NCT03457298 (Dowlatshahi et al., 2022)). At 9 months, the FDBA/Bio-Gide® group exhibited significantly greater gains in horizontal ridge width (0.95 ± 0.2 mm at −2 mm) and an increase in overall augmented site volume (from 266 ± 149 mm^3^ to 360 ± 138 mm^3^), whereas treatment with Ossix Volumax® resulted in minimal changes. These findings suggest that the osteogenic activity of collagen matrices may be insufficient to compete with native decellularized bone graft materials, which remain the current gold standard for alveolar ridge augmentation (Amini and Lari, 2021; Zubery et al., 2008).

With respect to oral soft tissue regeneration, several studies have evaluated the clinical feasibility of collagen matrices in the treatment of localized gingival recessions and root coverage. In a randomized clinical trial assessing combinations of coronally advanced flap (CAF) surgery with enamel matrix derivative (EMD) and/or the collagen matrix Mucograft®, complete root coverage was primarily influenced by EMD application, whereas a significant increase in keratinized tissue thickness was observed only in treatment groups receiving Mucograft® (NCT02456337 (Sangiorgio et al., 2017)). Consistently, another study demonstrated that the adjunctive use of Mucograft® with CAF resulted in significantly greater keratinized tissue width (3.02 mm vs. 2.64 mm) and gingival thickness (0.52 mm vs. 0.27 mm) compared with CAF alone (NCT00902876 (Stefanini et al., 2016)). Similarly, PriMatrix® applied for root coverage under a coronally positioned flap after 6 months reduced the initial vertical recession depth by a mean of 86%, while complete root coverage was achieved in 66% of treated defects. However, due to the small sample size (n = 8) and absence of a control group, these data should be interpreted with caution and considered as preliminary results of clinical feasibility (Naud and Assad, 2020).

Nevertheless, autologous soft tissue grafting remains more effective for keratinized mucosa regeneration than collagen matrices as demonstrated in a study on the surgical treatment of peri-implantitis (NCT03551145 (Solonko et al., 2022)). However, patients favored the use of collagen matrices (Mucograft®) due to reduced postoperative pain and lower analgesic consumption, which is attributed to the absence of traumatic graft harvesting and the subsequent painful healing. To further enhance the regenerative performance of collagen membranes (Gintuit®), strategies such as seeding with keratinocytes have been explored. In one clinical study, this approach resulted in gingival regeneration with sufficient thickness (≥2 mm), as well as native color and texture, at 6 months post-treatment (NCT00587834 (McGuire et al., 2011)). Importantly, patients in this study also expressed a preference for collagen matrix implantation over free gingival grafts due to reduced morbidity and the elimination of a donor site.

### 4.3 Ophthalmology

Corneal injuries and diseases in severe cases require full or partial corneal replacement in millions of patients worldwide, while the available number of donor corneas is strictly limited (Barrientez et al., 2019). The application of artificial collagen-based corneas can meet this demand, but this treatment option requires thorough tuning of implant properties, which is considered a difficult technological challenge (Fagerholm et al., 2010).

It was reported that collagen scaffold for cornea regeneration must possess mechanical properties that mimic the natural cornea to ensure adequate surgeon handling, tissue support and healing (tensile strength ∼ 3–4 MPa, elongation at break ∼60%, Young’s modulus ∼ 3–16 MPa) (Rafat et al., 2023; Mirazul Islam et al., 2015). However, it was later admitted that the implantation of such mechanically tough artificial cornea may lead to scar formation, and mechanically softer implant may be required to avoid this side effect (Rafat et al., 2023). Having a thickness of 100–300 μm, which is comparable to the natural cornea’s stromal layer, the implant should have the sufficient transparency (>80%) ensuring the visual function (Rafat et al., 2023; Koulikovska et al., 2014; Lei et al., 2022). The implant should be stable and resistant to biodegradation (years after implantation), while not eliciting immune responses and neo-vascularization (Hackett et al., 2011; Fagerholm et al., 2014). However, the fast epithelization and nerve re-growth are required for functional corneal recovery and prevention of its opacity (Fagerholm et al., 2010).

Similar physicochemical characteristics are applied to intraocular lenses. Their optical transparency (>90–95%) should be accompanied with the refractive index close to that of natural crystalline lens (∼1.42) (Bhattacharjee et al., 2023; Borja et al., 2008). Since collagen hydrogels typically have lower refractive index (<1.4) and mechanical properties than natural tissue, their inclusion into IOLs may result in lower optical power and instability, which require additional modification and stabilization of collagen (Hayes et al., 2015). Besides, IOLs should possess long-term optical and biostability with resistance to biodegradation and calcification, which limits the use of potentially pro-calcifying cross-linkers, such as glutaraldehyde (Sinha et al., 2012; Mackert et al., 2021).

#### 4.3.1 Product information

Collagen medical devices for ophthalmic applications are predominantly composed of porcine-derived sources (Supplementary Table S1c). ABCcolla® is produced from porcine corneal tissue using supercritical carbon dioxide (scCO_2_) decellularization, whereas XENIA® is manufactured from decellularized porcine corneal tissue followed by compression and UV/actinic-radiation cross-linking. In contrast to these tissue-derived corneal matrices, LinkCor® and Visian® are produced using reconstitution-based approaches.

To achieve the functional and optical properties, manufacturers may incorporate additional components into the membrane composition. Notably, Visian® is fabricated from collamer, a multicomponent collagen-based material designed to provide ultraviolet (UV) protection while maintaining optical transparency (Oral and Peppas, 2006; Marcinkowska et al., 2019; Feingold and Osipov, 1999).

The mechanical characteristics are improved by cross-linking. Radiation-induced cross-linking activating polymerization processes is applied in Visian®. As well, zero-length cross-linking with 1-ethyl-3-(3-dimethylaminopropyl)carbodiimide/N-hydroxysuccinimide (EDC–NHS) chemistry may be combined with UV-cross-linking following riboflavin pre-treatment, which has been reported to improve mechanical strength, optical transparency, and bioresorption times as exemplified by LinkCor® (Rafat et al., 2023).

#### 4.3.2 Clinical performance

In several clinical trials (NCT04653922, NCT04054817, NCT04741230) LinkCor®, ABCcolla®, and XENIA® have been investigated as corneal implants for the treatment of keratoconus. However, published data are currently available only for LinkCor®.

At 24 months post-implantation in 20 patients with keratoconus, LinkCor® ensured a significant increase in corneal thickness (200–285 μm) and a reduction in maximum keratometry values (11–14 D). Moreover, among 14 patients who were initially classified as legally blind, a final mean best-corrected visual acuity of 20/36 was achieved, along with restored tolerance to contact lens wear, highlighting the potential of LinkCor® to restore functional vision (NCT04653922) (Rafat et al., 2023).

Similar results were achieved in a study by Jabarvand et al. (Jabarvand et al., 2026), where 12 months after LinkCor® implantation in 17 patients, the central corneal thickness increased from 417 to 698 μm, anterior K_max_ decreased by 9 D, as well as best-corrected visual acuity improved, with no serious intraoperative or postoperative complications. Nevertheless, longer-term follow-up studies are required, as donor corneal transplantation may provide superior visual outcomes beyond a four-year period compared to collagen implant, which was demonstrated in the previous version of LinkCor® with another manufacturing technology (Fagerholm et al., 2014).

For XENIA®, the relevant clinical evidence is insufficient, since it is based on seven cases (El-Massry et al., 2021). At 12 months, despite the improvement in visual acuity, the central corneal thickness increased only by 40 μm, while K_max_ decrease was not statistically significant. In addition, one implant clouded and wrinkled, which required its replacement after 3 months. Furthermore, the interpretation of the XENIA® efficacy is limited, since after 3 months post-implantation 6 of 7 eyes underwent corneal cross-linking (CXL) due to progression of keratometric readings, thus confounding the effect of the implant with adjunctive CXL.

The direct comparison of LinkCor® and XENIA® clinical performance is not possible due to varying clinical protocols. However, these results should be matched with *in vitro* collagenase resistance tests performed for XENIA® and LinkCor®. XENIA® lenticules showed only 19.2% ± 13.7% area digestion after 20 days in collagenase solution (0.1 U/mL) (Reiter et al., 2025), while LinkCor® was tested under collagenase activity of 5 U/mL, and it reached 50% weight loss after 24 h (Rafat et al., 2023). For further artificial cornea development, the researchers should establish a standardized *in vitro* testing protocol for collagenase resistance, and compare these results with clinical data. This will significantly facilitate the comparison of results from new artificial cornea developers with the limited but highly valuable data on implants that have reached the clinical translation phase.

An additional noteworthy study evaluated the treatment of keratoconus using a combined approach involving implantation of a Visian® intraocular lens and corneal cross-linking. After 4 years of follow-up, patients reported significant improvements in visual acuity and a reduction in refractive errors (NCT02833649 (Doroodgar et al., 2017)). However, postoperative elevation of intraocular pressure remains an important concern associated with intraocular lens implantation, and it should be carefully considered during preoperative planning (Gong et al., 2024).

### 4.4 Orthopedics

Collagen membranes are used as adjunctive materials for the repair of rotator cuff injuries. The required mechanical properties of these scaffolds depend on their intended function. Scaffolds designed for mechanical reinforcement should provide sufficient strength and suture retention to support the repaired tendon during the early healing period, which is particularly important during the first 12 weeks after surgery. The mechanical properties of the native rotator cuff, including stiffness of approximately 200 N/mm and ultimate load of approximately 800 N, have been proposed as target values for the development of mechanically reinforcing scaffolds (Ricchetti et al., 2012). However, these values represent the properties of native rotator cuff tendon and should not be considered “ideal” general mechanical requirements for collagen scaffolds. Alternatively, some researchers consider the bioinductive function of collagen matrix to be the key parameter influencing the rotator cuff regeneration (Thon et al., 2020). In this case, the scaffold should maintain sufficient structural integrity for surgical handling and fixation while supporting cellular infiltration, vascularization and collagen deposition at the repair site (Longo et al., 2025).

#### 4.4.1 Product information

The collagen membrane REGENETEN® is manufactured from a solution of type I atelocollagen obtained by pepsin digestion of bovine tendons (Supplementary Table S1d). The resulting collagen solution is subsequently lyophilized to produce the final membrane structure. In 2016, the 2^nd^ version of REGENETEN® implant was announced (US20240374369A1 (Barnes et al., 2022)), where collagen was reinforced with a higher-strength reinforcement layer (including decellularized dermal graft or synthetic woven poly-L-lactic acid mesh). However, we found no evidence of its clinical application yet.

A variety of products are manufactured using decellularization of collagen-rich tissues, including porcine peritoneal tissue, as in CelGro® (Orthocell); SIS, as in Restore® (porcine source) and CuffPatch® (porcine source); the skin dermal layer, including GraftJacket® (human source), ArthroFLEX® (human source), Conexa® (porcine source), TissueMend® (bovine source), Zimmer® Collagen Repair/Permacol® (porcine source); as well of pericardium, such as in OrthADAPT® Bioimplant® (equine source) (Ricchetti et al., 2012).

CelGro® has a tri-laminar architecture consisting of a porous core between surfaces with different collagen fiber organization. This architecture provides a bioinductive matrix for tendon-derived cell attachment and growth with sufficient mechanical strength and suture retention for onlay fixation (Chen et al., 2021).

#### 4.4.2 Clinical performance

The effectiveness of collagen membranes in rotator cuff repair remains controversial and depends heavily on the specific product. REGENETEN® was effective in the treatment of full-thickness rotator cuff tears in a study by Bushnell et al. (Bushnell et al., 2021). In 115 patients, the improvement in shoulder function scores at 1 year follow-up was demonstrated, which was confirmed by magnetic resonance imaging (MRI) investigation. In a study by Yoo et al. (Yoo and Lee, 2025), REGENETEN® was applied as an onlay implant for isolated bursal-sided partial-thickness tears. This approach resulted in a reduced pain by 2 weeks and improvement in the shoulder function from 6 weeks onward, with no graft failures at 12 months. However, both studies lacked control group.

In NCT04444076 augmentation with REGENETEN® in medium-to-large full-thickness posterosuperior rotator cuff tears significantly reduced retear rates at 12 months from 25.8% in the control group (no augmentation) to 8.3% in the REGENETEN® group according to MRI investigation (Ruiz et al., 2024). Conversely, in the randomized study on large or massive full-thickness tears (NCT04450342), REGENETEN® augmentation showed no reduction in retear rates 6 months post-surgery compared with standard arthroscopic rotator cuff repair: 57.1% vs. 50.0%, respectively. The study was terminated early for reasons unrelated to safety. To better analyze REGENETEN® clinical and economic value, NCT06857084 was initiated by Smith and Nephew with follow-up period up to 24 months.

The bioinductive properties of CelGro® were investigated using human primary tendon-derived cells. The scaffold supported cell attachment and proliferation, while application of 6% uniaxial mechanical loading increased cell growth and expression of tendon-related markers, including TNMD, tenascin-C, Mohawk, and collagen-1α1. These results indicate that the aligned collagen architecture may support tendon cell differentiation and matrix formation under mechanical stimulation (Chen et al., 2021).

CelGro® was evaluated as an onlay scaffold for augmentation of rotator cuff repair in a prospective single-arm study involving 18 patients with tears >20 mm. At 12 months, MRI demonstrated complete healing in 64.8% of patients, while partial- and full-thickness retears were each observed in 17.6% of cases. Shoulder function also improved, with ASES scores increasing from 61.1 to 91.0 and Constant-Murley scores from 51.4 to 83.6, while VAS pain scores decreased from 5.6 to 0.9. No revision surgeries were required (Chen et al., 2021).

Implants made from decellularized human skin have also shown mixed results. In a study using GraftJacket® for bridging reconstruction of large or massive irreparable tears, the rate of retears was substantially lower than after maximal repair alone: 21% vs. 87%, respectively (NCT01987973 (Karpyshyn et al., 2025)). In another study (Cutbush et al., 2025), allografts ArthroFLEX® and GraftJacket® were applied for combined cuff repair and superior capsular reconstruction reinforcement. At a mean of 14.4 months, MRI showed 6 isolated capsular reconstruction failures and 5 isolated rotator cuff retears among 50 patients, which is a promising result considering that before operation, 14 patients already =had previous failed rotator cuff repair. For ArthroFLEX®, no other published clinical results were identified: NCT06215417, NCT03511547, and NCT03551509 were ongoing or discontinued.

On the other hand, in NCT04975581, the use of collagen membranes led to poorer outcomes in studies with small patient samples: early biopsy analysis showed that both GraftJacket® (human nature) and Permacol® (xenogenic nature) disrupted the native supraspinatus extracellular matrix 4 weeks post-implantation (Rashid et al., 2020), while more pronounced immune response was observed in Permacol® group. Furthermore, Soler et al. (Soler et al., 2007) reported Permacol® (Zimmer® Collagen Repair Patch) graft failure in all 4 patients with massive tears within 3–6 months period, which involved: graft disintegration, tissue necrosis, inflammatory reaction, and retears.

However, Zimmer® Collagen Repair Patch demonstrated positive results in small uncontrolled studies on massive rotator cuff tears. MRI showed preserved repair integrity in 8 of 10 cases after 3–5 years (Badhe et al., 2008) or in 4 of 5 cases at 8-month period (Cho et al., 2014), while in both studies, the reduction in shoulder pain was reported by patients.

Conexa® also showed mixed results. In NCT01025037, Conexa® was used for reinforcement of large rotator cuff tears in 61 patients. Despite the improvement in functional scores and strength, MRI showed retears in 33.9% of patients (NCT01025037 (Lederman et al., 2016)). Furthermore, in a prospective comparative study of large-to-massive tears, Conexa® augmentation showed no advantage over standard repair procedure and led to more complications (Maillot et al., 2018). A subsequent letter to the editor affiliated with Stryker noted that this negative result should be interpreted cautiously since the study was non-randomized, underpowered, and probably included more complex tears in the patch group (Dimock and Narvani, 2019).

TissueMend®, Restore® and CuffPatch® did not achieve broad clinical adoption, probably due to weak mechanical properties (Barber et al., 2006). In identified human studies, Restore® failed to improve healing after augmentation (Iannotti et al., 2006), as well as caused early inflammatory reactions, a high retear rate or demonstrated no recognizable benefit compared with controls (NCT00208338 (Malcarney et al., 2005; Sclamberg et al., 2004; Walton et al., 2007)). As well, pericardium-derived OrthADAPT® implant showed unfavorable safety results in full-thickness rotator cuff tears (Eudra-CT 2007–007630-19 (Lamas et al., 2019)) despite the high theoretical biocompatibility of equine collagen sources (Rezaeivandchali et al., 2026).

Thus, collagen membranes demonstrated the ability to improve tissue healing in selected rotator cuff tear cases, but their clinical performance should be further elucidated depending on tissue source, material architecture, cross-linking, mechanical strength, degradation kinetics, and immune response. Further enhancement of the therapeutic efficacy of collagen membranes in rotator cuff repair may be achieved through the incorporation of cell-based strategies. A meta-analysis of preclinical studies suggests that such approaches can improve both biomechanical properties and functional clinical scores (Morton-Gonzaba et al., 2020).

## 5 Sponges

### 5.1 Dentistry

Collagen devices are used to repair bone defects, particularly, alveolar defects resulting from tooth extractions, periodontal diseases, and trauma. For optimal bone repair, a collagen sponge should provide mechanical strength and mineral components, as well as be porous to promote cell migration and differentiation thus becoming an osteoconductive material (Yamada and Egusa, 2018). The optimal pore size for the sponges is 100–400 μm, while various complications may occur at smaller pore sizes: pore sizes of 40–100 μm may result in osteoid, pore sizes smaller than 40 μm lead to the formation of fibrovascular capsule (Sammarco and Chang, 2002). The sponge should also have a hemostatic effect, since alveolar defects are usually accompanied by bleeding (Le et al., 2018). To provide osteoinductive properties, various biologically active substances may be added to the sponge: platelet-derived growth factor (PDGF), transforming growth factor-beta (TGF-β), recombinant human bone morphogenetic protein (rhBMP-7, rhBMP-2), and fibroblast growth factor (FGF) (Le et al., 2018).

#### 5.1.1 Product information

Bovine (Hemocollagene®, NuOss XC®, Osteogen Plug®) and porcine (Bio-Oss® Collagen) sources of collagen are used to produce the sponges (Supplementary Table S2a). The main technology for their production is lyophilization, which ensures highly porous structure (Katrilaka et al., 2023). In order to improve the osteoconductive properties of collagen sponges, the manufacturers apply mineral components: calcium hydroxyapatite (Ossix Bone®, 80% wt.), a special form of calcium apatite (Osteogen Plug®), as well as anorganic xenogenic bone granules (Bio-Oss Collagen® 90% wt., NuOss XC® 80% wt.). To enhance the mechanical properties, the sponges may be cross-linked, for example, with GLYMATRIX® technology (Ossix Bone®), which is based on ribose-induced cross-linking of collagen that mimics glycation (Monstrey et al., 2007).

#### 5.1.2 Clinical performance

Bio-Oss Collagen® implantation after a tooth extraction procedure was effective in reducing the horizontal alveolar changes at 4 months in NCT03422458 (Clementini et al., 2019). Similar results were shown in NCT03395145 (Gabay et al., 2022) at 6 months after implantation, however, a slow degree of resorption was noted for the implant mineral component. The newly formed bone in the Bio-Oss Collagen® group constituted 33.79% ± 17.37% of the original defect, while in the control group (spontaneous healing), it constituted 51.14% ± 23.04%. Similarly, the aspect of Bio-Oss Collagen® slow resorption in the extraction socket was demonstrated in NCT03659617 (Couso-Queiruga et al., 2023), which was probably due to the high mineral content in the implant (90% wt.) (Liao and Cui, 2004; Salgado et al., 2016). Indeed, when using Ossix Bone® implants with a lower mineral content, they showed a higher volume of vital bone formation in the socket after 4 months compared to Bio-Oss Collagen®: 39.3% ± 17.8% vs. 26.8% ± 15.8, respectively (NCT04338516 (Casarez-Quintana et al., 2022)).

The problem of balance between bone formation and graft resorption was also observed for OsteoGen Plug® in alveolar ridge preservation after tooth extraction. In NCT05419778, OsteoGen Plug® showed no statistically significant difference in bone area compared with allograft bone chips covered by a non-resorbable PTFE membrane at 12–20 weeks post-implantation: 35.89% vs. 43.87%, respectively. However, the residual graft material was found in all OsteoGen Plug® samples, and foreign-body granulomatous inflammation was observed in 3 of 6 cases (Jafarian et al., 2024).

On the one hand, the slow resorption rates can enhance the long-term stability of the implant and ensure the bone volume preservation, which is critical for further surgical procedures​ (Moreira et al., 2019). On the other hand, the remaining inorganic components can inhibit cell migration, new tissue ingrowth, and the vasculature formation necessary to initiate reparative processes, which may have negative consequences such as graft material displacement, oro-antral and oro-nasal communications, implant failure, foreign body reactions, soft tissue fenestrations and associated cysts (Rodríguez and Hessam, 2020). One possible option to accelerate the implant degradation is to mix its main inorganic component hydroxyapatite with β-tricalcium phosphate (β-TCP), which will also enhance the osteoconductive and osteoinductive properties (Yamada et al., 1997; Jacob et al., 2023).

### 5.2 Ophthalmology

Collagen sponges are applied for trabeculectomy, an operation to lower intraocular pressure (IOP) in patients with glaucoma by excising the part of the eye’s trabecular meshwork (Elwehidy et al., 2024). During the surgery, the implant is placed on the scleral flap and covered with conjunctiva, forming a bubble (bleb). The use of collagen sponge during trabeculectomy helps to reduce potential complications, such as hypotony or improper bleb formation (Zelefsky et al., 2008; Gack and Hohberger, 2021; Realini et al., 2021).

The sponge modulates aqueous humor flow by maintaining pressure and acting as a reservoir for the absorbed aqueous humor, thereby protecting the eye from elevated pressure and preventing hypotony (He et al., 2014). Specifically, the sponge ensures the formation of the blebs with proper morphology, which is essential for the success of trabeculectomy (Leung and Tham, 2013). Being porous, it guides fibroblast ingrowth in a loose way rather than in an organized way, which influences tissue remodeling processes and prevents scar formation (Boey et al., 2010). In addition, by mechanically separating the episcleral surface from the conjunctiva, the implant facilitates proper bleb formation by occupying the subconjunctival space and preventing adhesion formation (Wlaź et al., 2019).

#### 5.2.1 Product information

We found only one product used for trabeculectomy (Ologen®) (Supplementary Table S2b). It is a sponge composed of porcine atelocollagen type 1 (90%) and glycosaminoglycan, and is produced by freeze-drying. The sponge has a porous structure with a pore diameter of about 10–300 μm (Cho and Lee, 2015). After the implantation, the device completely degrades within 90–180 days (Zelefsky et al., 2008; Cillino et al., 2011).

#### 5.2.2 Clinical performance

Generally, in the studies comparing the efficacy of Ologen® implant and mitomycin C (MMC) application after trabeculectomy, no significant differences were reported in terms of success rates between the groups. However, in NCT03506542 (Wlaź et al., 2019), during the first 3 months, the bleb height score was significantly greater in the Ologen® group than in the MMC group, which potentially may contribute to early bleb leakage and lead to transient postoperative hypotony. From the other side, the MMC group in NCT01440751 (Tanna et al., 2016) experienced persistent hypotony compared to the Ologen® group (18.8% vs. 0%), which makes the latter treatment option more favorable for certain patient groups.

Ologen® may also be used to reduce the visibility of blue sclera discoloration after strabismus surgery, as was shown in a study (NCT02699580 (Yoo et al., 2017)) in comparison to standard treatment group. However, the study had a short follow-up period, and in the longer period, the observed differences could mitigate.

As well, Ologen® may be applied in combination with antifibrotic agents to improve control of postoperative scarring. In a retrospective study in which all patients underwent mini-shunt implantation, 12-month post implantation, Ologen® combined with 5-fluorouracil was associated with lower intraocular pressure, fewer reoperations, and bleb leakage compared to blank Ologen® group (Menda et al., 2015). However, the adjunction may not always be beneficial. Min et al. reported that Ologen®, when soaked in MMC, increased the generation of the encapsulated blebs (Min et al., 2013). It was suggested that this effect may occur due to local inhibitory effect of MMC on the ingrowing of fibroblasts, while causing their excessive proliferation around the implant thus provoking the bleb encapsulation (Wlaź et al., 2019).

Further investigations into Ologen® efficacy and safety are required to fully understand its role and potential benefits over traditional techniques and materials used in ophthalmic surgery.

### 5.3 Dermatology

A variety of skin grafts are currently available for wound management, including autografts and allografts. However, due to several inherent limitations, such as limited donor availability, traumatization, risk of disease transmission, and immunogenicity, the clinical use of collagen-based artificial skin substitutes has gained increasing attention (Tian et al., 2024).

To provide the moist wound environment required for effective skin regeneration, collagen-based wound dressings must exhibit an appropriate moisture vapor transmission rate, typically within the range of 2000–2500 g/m^2^/24 h (Xu et al., 2016). This range allows excess moisture to evaporate while maintaining adequate hydration at the wound site without leakage (Queen et al., 1986). In addition, the maintenance of a moist environment is supported by the water-retention capacity of the dressing. Accordingly, the swelling or absorptive capacity should not be less than 150% (i.e., at least 1.5 g of fluid per 1 g of material) (Minsart et al., 2022).

The thickness of the wound dressing should correspond to that of the damaged skin layer, most commonly ranging from 1 to 6 mm, to ensure close conformity to the wound bed and to minimize the formation of dead spaces that may serve as foci for infection (Yoon et al., 2016; Yannas and Burke, 1980; Uzun et al., 2013).

Porosity represents another critical parameter of collagen-based sponges, as it facilitates cell adhesion and supports cellular proliferation. Current studies indicate that an optimal pore size of collagen scaffolds that promotes wound healing while minimizing wound contraction and providing favorable conditions for tissue regeneration lies within the range of 20–120 μm (Yannas et al., 2010).

In addition to pore size and overall porosity, the spatial organization of the pore network is increasingly recognized as an important determinant of scaffold performance. The architecture of collagen sponges is established during the freeze-drying process, and it is governed by multiple manufacturing parameters, including slurry composition and viscosity, freezing conditions, gelation, and post-lyophilization processing (Diaz et al., 2023). By controlling these parameters, scaffolds with distinct pore morphologies, ranging from randomly interconnected to highly aligned architectures, can be produced, depending on the requirements for the specific clinical application (Figure 3).

**FIGURE 3:**
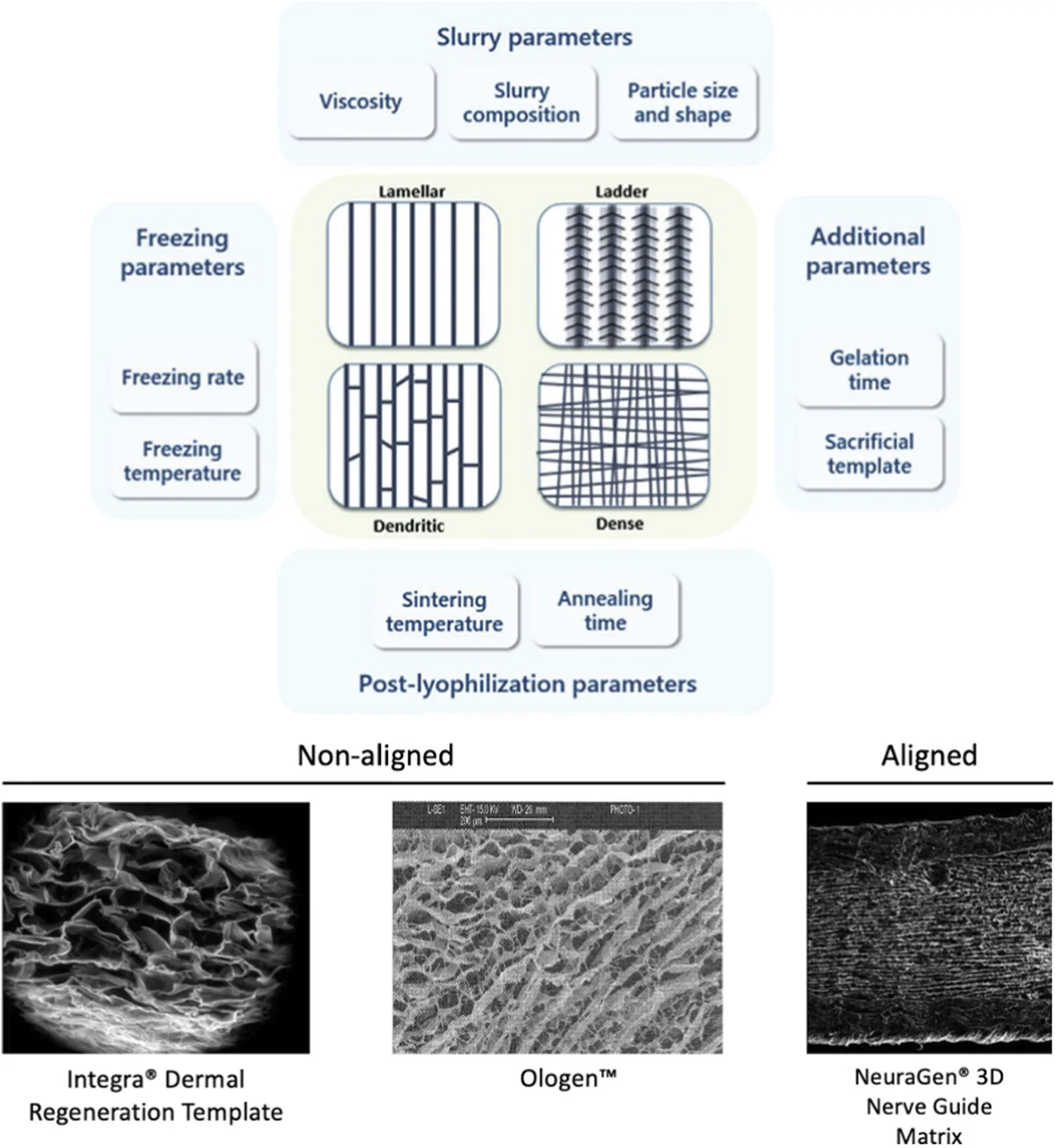
Key processing variables and resulting pore morphology in non-aligned versus aligned collagen scaffolds. Reproduced with the permission from (Sagentia, n.d.; Diaz et al., 2023; Integra® Dermal Regeneration Template n.d, 2026; Tu et al., 2002).

#### 5.3.1 Product information

Examples of products produced by the decellularization of porcine tissues are PuraPly® and OASIS® Wound Matrix (SIS), while matrix derived from native axolotl tissue is represented by NeoMatriX® (Supplementary Table S2c). The latter is obtained through repeated exposure to hypo- and hyperosmotic sodium chloride solutions followed by lyophilization.

Other products are manufactured using reconstitution approaches. For instance, Integra® incorporates enzymatically and alkali-treated bovine tendon collagen, whereas Helicoll® is derived from bovine collagen treated with papain. Lyophilization is commonly used to obtain the final product (PROMOGRAN®, Integra®), although air-drying techniques have also been reported (Helicoll®).

To enhance functional properties, manufacturers incorporate additional components to collagen, such as oxidized cellulose (PROMOGRAN®), chondroitin-6-sulfate (Integra® Dermal Regeneration Template n.d, 2026), which is an extracellular matrix glycosaminoglycan known to play an important role in cell growth, differentiation, morphogenesis, and migration (Yamada and Sugahara, 2008). Furthermore, to improve resistance to proteolytic degradation by wound exudate, collagen is chemically cross-linked. Commonly employed cross-linking agents include EDC hydrochloride (PuraPly®) and glutaraldehyde (Integra® Dermal Regeneration Template n.d, 2026).

#### 5.3.2 Clinical performance

Collagen-based wound dressings have demonstrated favorable outcomes in the management of various wound types. In a clinical trial on mixed cutaneous wounds (venous leg ulcers, diabetic foot ulcers, post-surgical wounds, pressure ulcers), treatment with PuraPly® resulted in complete wound closure in 73% of patients within 32 weeks, with a median time to closure of 17 weeks (NCT03286452 (Bain et al., 2020)). In another study employing a similar therapeutic approach and comparison groups, a reduction in wound area was observed in 73% of patients within 12 weeks, while complete wound closure was achieved in 37% of patients over a mean duration of 6.7 weeks (NCT03070925 (Oropallo, 2019)).

NeoMatriX® has only preliminary clinical results published (Malueg et al., 2025). It was used for primary or secondary wound closure after neurosurgical procedures in 23 high-risk patients. The postoperative wound infection or dehiscence were observed only in 8.7% cases, and minimal scarring of implantation site was reported in all patients. A notable limitation of the listed studies on PuraPly® and NeoMatriX® is the absence of standard-of-care control groups, which restricts the strength of comparative conclusions.

More robust comparative evidence is available for OASIS® Wound Matrix. In full-thickness diabetic foot ulcers, OASIS® achieved complete closure in 49% of patients at 12 weeks compared with 28% in the Regranex® group (becaplermin gel with topical recombinant human platelet-derived growth factor-BB) (Niezgoda et al., 2005). In chronic leg ulcers, OASIS® combined with compression therapy increased healing rates from 34% (standard-care group) to 55% at 12 weeks (Mostow et al., 2005). In difficult-to-heal mixed arterial/venous ulcers, OASIS® also was associated with a higher closure rate than Hyaloskin® (hyaluronic acid-derived extracellular matrix), 82.6% vs. 46.2%, respectively (Romanelli et al., 2007). In stage III–IV pressure ulcers, complete closure at 12 weeks was numerically higher for OASIS® than for standard care, 40% vs. 29%, respectively, while, however, the difference was not statistically significant (Brown-Etris et al., 2019).

In diabetic foot ulcers, the evidence for other collagen-based wound dressings remains heterogeneous. PROMOGRAN® achieved complete closure in 37.0% of patients at 12 weeks compared with 28.3% in the moistened gauze group, but the overall difference was not statistically significant (Veves et al., 2002). Helicoll® showed faster early healing in a small randomized study, with 50% wound closure by 4 weeks compared with 25% in the amnion/placenta-derived comparator group, but the short follow-up period and limited sample size restrict study interpretation (NCT06557122 (Gunasekaran, 2026)). By contrast, Integra® demonstrated a stronger effect in diabetic foot ulcers, increasing the complete closure rate compared with standard care at 12 weeks, 51% vs. 32%, respectively (NCT01060670 (Driver et al., 2015)). It was also reported to outperform decellularized allogeneic human skin (DermaCELL®) in deep diabetic foot ulcers (NCT03476876).

Across the analyzed studies, the least favorable skin regeneration outcomes were observed in diabetic ulcers and pressure ulcers. These wound types are primarily driven by impaired innervation and compromised blood supply, pathophysiological factors that collagen-based matrices can address only to a limited extent (Huang and Kyriakides, 2020).

### 5.4 Gastroenterology

Two principal applications of collagen devices have been identified in this field. The first involves implantation onto the pancreatic stump for the prevention of clinically relevant postoperative pancreatic fistula following distal pancreatectomy. Currently employed techniques, such as stapling and sealing, have demonstrated limited efficacy. Consequently, the use of collagen-based materials has attracted increasing interest. These materials are proposed to reduce pancreatic juice infiltration through mechanisms including bioadhesion, hemostatic activity, and resistance to proteolytic enzymes, thereby facilitating tissue healing (Chaouch et al., 2024; Chattopadhyay and Raines, 2014; Serradilla-Martín et al., 2023; Reeh et al., 2011; Nahm et al., 2018).

The second application area concerns the treatment of parastomal hernias. A variety of surgical approaches have been described, including stoma relocation and reinforcement with mesh implants composed of synthetic or biological polymers (Colvin and Rosenblatt, 2018). Biological materials are generally considered advantageous due to their ability to support revascularization and tissue integration, as well as their lower susceptibility to infection, which contributes to their preferential use in certain clinical settings (Nahabedian et al., 2018). The primary function of such implants is to enhance the mechanical integrity of the abdominal wall. Accordingly, key requirements for collagen sponges in this context include high mechanical strength and a prolonged biodegradation period (Pascual et al., 2012). The target tensile strength of the biomaterial should be no less than 16 N/cm (Klinge et al., 1998), with an elongation at this load of 15% (Bachman and Ramshaw, 2008), as calculated according to established testing protocols (Klinge et al., 2003).

#### 5.4.1 Product information

To preserve native mechanical properties and biological activity of the collagen matrix, decellularization followed by drying of minipig dermis was applied to produce Strattice® (Supplementary Table S2d).

The incorporation of bioactive components into collagen-based devices determines their mechanism of interaction with biological tissues. Depending on the formulation, tissue adhesion is achieved either through covalent bonding mediated by reactive polymers or through the formation of a fibrin clot following activation of the coagulation cascade. These distinct mechanisms are schematically illustrated in Figure 4.

**FIGURE 4:**
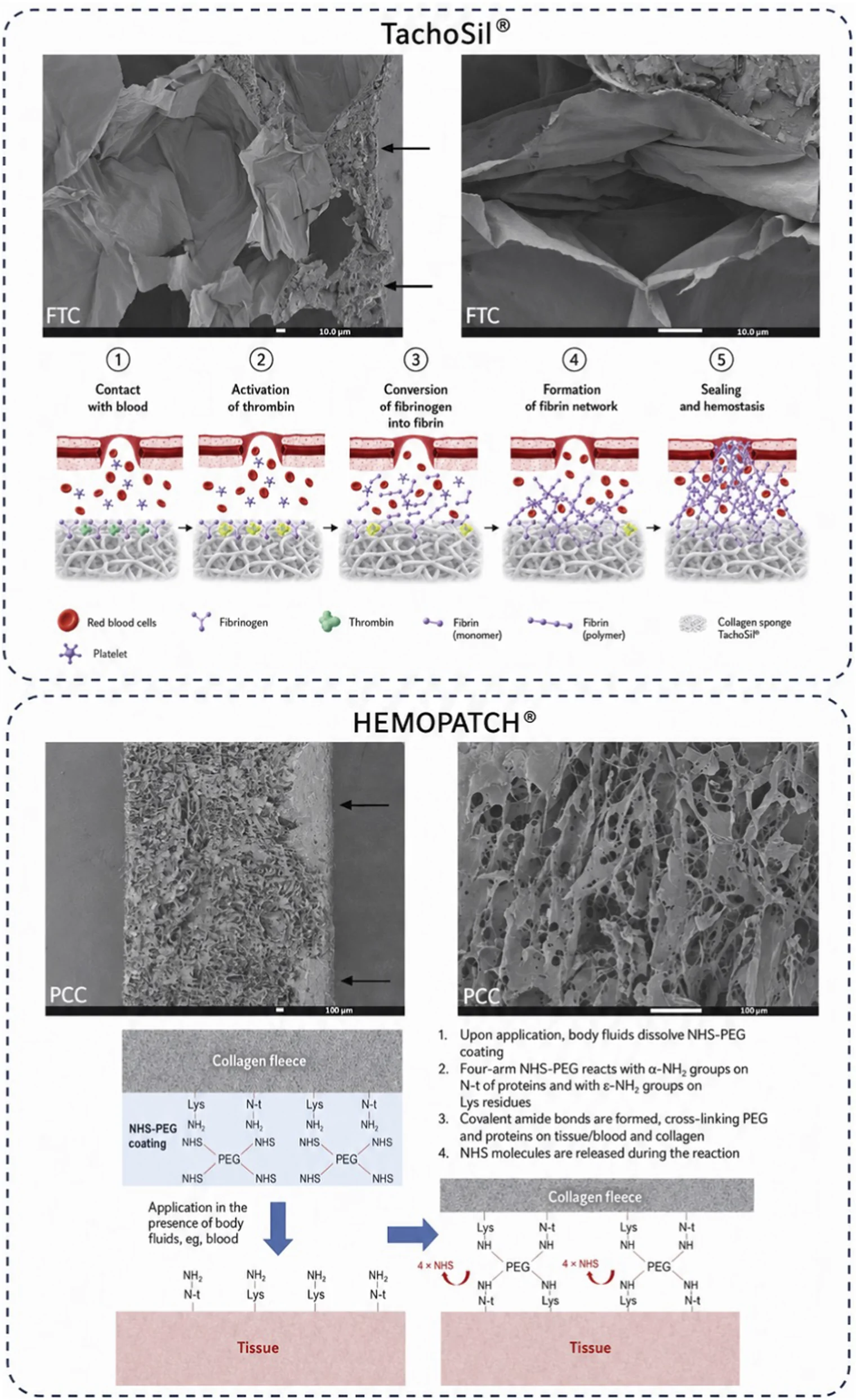
Comparison of the structural features and mechanisms of action of TachoSil® and HEMOPATCH®. Upper panel: TachoSil® consists of a collagen sponge coated with human fibrinogen and thrombin. Lower panel: HEMOPATCH® is composed of a collagen sponge coated with N-hydroxysuccinimide polyethylene glycol (PEG-NHS). Representative scanning electron microscopy (SEM) images illustrate the microstructure of each collagen sponge. Arrows indicate the external surface (coated side) of the sponge. Reproduced with the permission from (Baumgartner et al., 2016; Lewis et al., 2015).

Fibrinogen and thrombin are incorporated into the structure of TachoSil®. These components interact with blood constituents to form a fibrin clot, thereby ensuring the fixation and tissue adhesion of the device (Risman et al., 2024). HEMOPATCH® contains 4-arm polyethylene glycol N-hydroxysuccinimide (4-arm PEG-NHS) and COH206 4-arm polyethylene glycol thiol (4-arm PEG thiol). Upon interaction, these components form a stable hydrogel network that promotes strong adhesion to adjacent tissues (Hedrich et al., 2011). It is speculated that PEG-NHS coating provides an enhanced hemostatic effect by forming a hydrogel that is more resistant to enzymatic degradation by pancreatic enzymes compared with fibrin-based sealants (Serradilla-Martín et al., 2023).

#### 5.4.2 Clinical performance

In a clinical study, the use of TachoSil® patches for the prevention of postoperative pancreatic fistula (POPF) following distal pancreatectomy did not demonstrate a statistically significant benefit compared with the control group, with clinically relevant POPF occurring in 22.9% and 28.3% of patients, respectively (NCT01550406 (Park et al., 2016)). Similarly, another study found no reduction in POPF incidence with the use of HEMOPATCH® (25%) when compared with the historical baseline rate of POPF (20%) observed in non-augmented cases (NCT03410914 (Bubis et al., 2022)). The lack of a measurable protective effect has been attributed, at least in part, to the inability of collagen matrices to fully seal the defect due to their inherent porosity and susceptibility to degradation by pancreatic proteases (Aprahamian et al., 1992).

The application of the collagen mesh Strattice® for the prevention of parastomal hernia did not demonstrate a clear advantage, either when compared with no reinforcement (24-month recurrence rates of 12.2% vs. 13.2%, respectively) (NCT00771407 (Fleshman et al., 2014)) or when compared with synthetic mesh alternatives (24-month recurrence rates of 29.8% vs. 25.5%, respectively) (NCT02451176 (Miller et al., 2022)). These findings suggest that the routine use of collagen meshes may not be justified in light of their high cost, and their application should be attributed to specific clinical scenarios where biological properties of collagen represent specific advantages (Köckerling et al., 2015).

### 5.5 Neurosurgery

The products are generally utilized as dural substitutes for the repair of dura mater defects, with the primary objective to prevent cerebrospinal fluid (CSF) leakage following surgical interventions (Fengbin et al., 2015; Esposito et al., 2016). US Food and Drug Administration (FDA) states that CSF leakage is a key clinical efficacy criterion for comparing different dural substitutes (U.S. Food and Drug Administration, 2026a). In terms of mechanical properties, the material should closely resemble those of the dura mater, which will ensure its resistance to surgical manipulations (tension and suturing) without tearing (Narotam et al., 2007). The following mechanical properties have been published for human temporal dura mater: elastic modulus of 70 ± 44 MPa, tensile strength of 7 ± 4 MPa, and maximum strain of 11% ± 3% (Zwirner et al., 2019).

#### 5.5.1 Product information

The density of the product material, necessary to ensure sealing effect, is achieved through a decellularization process that preserves the original tissue architecture, as in the Lyoplant®, which is derived from bovine pericardium (Supplementary Table S2e). The principal technique for generating the final implant architecture and porosity for tissue integration is lyophilization (HEMOPATCH®, Lyoplant®).

#### 5.5.2 Clinical performance

Collagen-based implants have demonstrated favorable safety and performance profiles. In one clinical study, the Lyoplant® onlay implant was associated with a CSF leakage rate of 6.6%, with no reoperations required for CSF leakage, indicating both safety and clinical effectiveness. Furthermore, handling characteristics were rated as “excellent” or “very good” by 96% of surgeons (NCT02678156 (Greifzu et al., 2022)). Similarly, HEMOPATCH® was evaluated in another study and demonstrated a comparable CSF leakage rate of 7.5%, further supporting its effectiveness in dural repair (NCT03392662 (Lombardo et al., 2022)). These data indicate that collagen-based implants can be successfully applied in dural reconstruction, achieving relatively high rates of surgical success while maintaining a low risk of procedure-related complications. However, the available evidence is largely limited to non-comparative studies.

### 5.6 General surgical applications

The primary purpose of this product group is to achieve effective hemostasis following various surgical dissection procedures, including the resection of parenchymal organs. The essential performance requirements for such medical devices are largely similar with those listed previously in gastroenterology and neurosurgery sections and include: sufficient mechanical strength, pronounced hemostatic and sealing properties (Maisano et al., 2009; Teoh et al., 2022).

In standardized preclinical evaluation, the hemostatic efficacy of collagen sponges is typically assessed in *in vivo* models (e.g., liver, kidney, or rabbit ear bleeding models), as they better reproduce physiological coagulation conditions compared to *in vitro* assays. In such models, effective materials are generally expected to achieve bleeding control within several minutes (commonly < 3–5 min), with advanced formulations demonstrating significantly shorter hemostasis times relative to gauze or conventional gelatin sponges (Li et al., 2026; Ding et al., 2024).

In addition to coagulation time, fluid absorption capacity is a critical functional parameter, as rapid uptake of blood promotes clot formation via concentration of platelets and coagulation factors. Collagen-based hemostatic sponges typically exhibit very high absorbency, with reported blood or fluid uptake ratios in the range of 2000–3500% of their own weight, depending on porosity and microstructure (Ding et al., 2024). Specifically, in the literature, swelling ratios around 2000% were associated with improved hemostatic performance (Wang et al., 2022).

#### 5.6.1 Product information

The medical devices are composed of bovine-derived collagen, as exemplified by HEMOPATCH® and Lyostypt® (Supplementary Table S2f). Adjunctive components may be included into the device, such as fibrinogen and thrombin (TachoSil®) and PEG-NHS (HEMOPATCH®), which contribute to hemostatic and adhesive properties of the collagen implant.

#### 5.6.2 Clinical performance

HEMOPATCH® (NCT03323359, NCT02133378, NCT04904653, NCT02633670), Lyostypt® (NCT00837954), and TachoSil® (NCT05385952) have been investigated as hemostatic agents in a variety of surgical applications. However, the results of these clinical trials have not been published to date.

TachoSil® demonstrated high efficacy as a hemostatic and sealing agent. In liver resection (NCT01192022), TachoSil® provided faster secondary hemostasis than oxidized regenerated cellulose gauze: 80.7% vs. 50.0% of patients achieved complete hemostasis within 3 min, and 94.7% vs. 76.4% within 5 min, respectively (Genyk et al., 2016). In pulmonary lobectomy (NCT00293514), TachoSil® reduced postoperative alveolar air leakage compared with standard management (resuturing, stapling or no additional treatment) by increasing intraoperative air-leak closure from 42% to 68% and shortening median leak duration from 19.5 to 14.5 h (Marta et al., 2010).

HEMOPATCH® has likewise shown favorable hemostatic performance, for example, in the case of partial nephrectomy compared with standard renorrhaphy, and its use was associated with a significant reduction in intraoperative blood loss (114 ± 159 ml vs. 146 ± 195 ml) and shorter operation time (43 ± 27 min vs. 53 ± 49 min) (Staehler et al., 2022). HEMOPATCH® has also been applied after axillary lymph node dissection with the aim of reducing the incidence of postoperative seroma formation. At 3 months of follow-up, clinically relevant seromas were detected in 6 patients (30%) in the HEMOPATCH® group compared with 18 patients (43%) in the control group (NCT04185480 (Spiekerman van Weezelenburg et al., 2022)).

Despite these generally positive outcomes, some studies and clinical observations raise concerns regarding the reliability of hemostatic performance of fibrinogen-coated collagen sponges. In particular, swelling of the fibrin layer on the surface of TachoSil® may impair the intrinsic absorbent capacity of the collagen matrix, thereby reducing its ability to effectively uptake blood and promote clot formation. Furthermore, questions remain regarding the long-term stability of adhesion at the bleeding site, as degradation of the fibrin layer may weaken the attachment of the sponge to tissue. The mechanical integrity of the fibrin-tissue interface itself has also been reported to be inferior compared to that achieved with certain synthetic adhesive layers. In this context, synthetic sealants based on reactive functional groups, such as NHS-esters, may provide stronger and more stable covalent bonding to tissue surfaces, potentially resulting in improved adhesion strength compared to fibrin-based solutions (Li et al., 2022a; Uslu et al., 2023).

### 5.7 Orthopedics

Collagen sponges are used in orthopedics as temporary porous matrices for ligament repair, bone regeneration, and articular cartilage restoration. Their high porosity and fluid absorption capacity enable the incorporation of autologous blood, cells, mineral components, or bioactive molecules, while providing a three-dimensional environment for cell infiltration and tissue formation (Glowacki and Mizuno, 2008). However, collagen sponges possess limited intrinsic mechanical strength and therefore cannot independently provide sufficient stabilization in load-bearing applications. Consequently, their clinical function is generally dependent on surgical fixation, combination with mechanically stable implants, or integration into composite constructs (Dong and Lv, 2016; Rico-Llanos et al., 2021).

In ligament repair, the collagen sponge should serve as a temporary bridge between the injured tissue ends. This function is particularly relevant to anterior cruciate ligament (ACL) injuries, as spontaneous ACL healing is impaired by the intra-articular environment. In contrast to extra-articular ligaments, a stable fibrin clot does not form between the ruptured ACL ends, which is associated with the thrombolytic environment of the synovial fluid (Dong and Lv, 2016; Murray and Fleming, 2013). A porous collagen scaffold saturated with autologous blood can retain the blood within the defect, provide a provisional extracellular matrix for cellular migration, and maintain contact between the torn ligament ends during the early stages of repair (Meaney Murray and Spector, 2001; Murray et al., 2019).

In bone regeneration, the limited osteoinductive properties of collagen sponges can be enhanced by their functionalization. Recombinant human bone morphogenetic protein-2 (rhBMP-2) can be incorporated into the collagen sponge to provide osteoinductive activity (Rico-Llanos et al., 2021; Geiger et al., 2003). Alternatively, mineral components, such as hydroxyapatite, can be incorporated to improve scaffold osteoconductivity (Li et al., 2021; Han et al., 2024).

Collagen sponges can also serve as three-dimensional cell carriers for articular cartilage regeneration. In matrix-associated autologous chondrocyte transplantation (MACT), the porous collagen matrix provides a three-dimensional environment for expanded autologous chondrocytes and enables their implantation into focal cartilage defects. Such constructs may additionally incorporate a denser collagen membrane layer to form a biphasic scaffold that combines cell retention within the porous matrix with structural coverage of the defect (Huang et al., 2016).

#### 5.7.1 Product information

Bovine type I collagen is used for the production of BEAR® Implant, INFUSE® (InductOs®), HEALOS® and NOVOCART 3D® (Supplementary Table S2g). Lyophilization remains the main technology for the production of the porous structure of these implants.

To provide the required biological properties, various components may be incorporated into collagen sponges. BEAR® is hydrated with up to 10 mL of autologous blood immediately before implantation. The blood is absorbed within the porous structure of the sponge, which is then positioned between the torn ACL ends and secured with sutures (Miach Orthopaedics, 2026). In INFUSE® (InductOs®), recombinant human bone morphogenetic protein-2 (rhBMP-2, dibotermin alfa) is applied to the collagen sponge at a concentration of 1.5 mg/mL to provide osteoinductive properties (INFUSE® Bone Graft Important Medical, 2026). Alternatively, mineral components may be incorporated into the collagen sponge to improve its osteoconductive properties, as exemplified by hydroxyapatite in HEALOS®. Before implantation, HEALOS® is additionally saturated with autologous bone marrow aspirate (Huang et al., 2016; HEALOS BONE GRAFT MATERIAL, 2025).

NOVOCART® 3D represents a cell-based functionalization strategy in which a collagen scaffold serves as a carrier for autologous articular chondrocytes. The implant has a biphasic architecture consisting of a dense, cell-impermeable collagen membrane derived from bovine pericardium and a porous collagen sponge, which are firmly connected by lyophilization. A distinctive feature of the sponge layer is its columnar, interconnected pore architecture oriented approximately perpendicular to the membrane, providing a three-dimensional matrix for chondrocyte distribution. Autologous chondrocytes isolated from a cartilage biopsy are expanded *in vitro* and seeded into the porous scaffold before implantation into the cartilage defect.

#### 5.7.2 Clinical performance

BEAR® demonstrated comparable clinical performance with autograft ACL reconstruction in the randomized study (NCT02664545). At 2 years, no significant differences were observed in the International Knee Documentation Committee (IKDC) subjective score (88.9 vs. 84.8) and anteroposterior knee laxity (1.61 vs. 1.77 mm), respectively. At the same time, BEAR® resulted in significantly higher hamstring muscle strength compared with ACL reconstruction (98.2% vs. 63.2% of the contralateral side), which was associated with the absence of tendon harvesting. However, subsequent ipsilateral ACL surgery was required in 14% of patients after BEAR® and 6% after ACL reconstruction, although this difference was not statistically significant (Murray et al., 2020). Similar results for BEAR® implant were demonstrated in the study NCT02292004 at six-year follow up. No significant differences between BEAR® and ACL reconstruction were observed in patient-reported outcomes and knee stability, while hamstring strength remained higher after BEAR® (Fleming et al., 2024).

INFUSE® has demonstrated favorable results in bone regeneration. In a randomized study on lumbar interbody fusion, the fusion rate at 24 months was 94.5% for INFUSE® compared with 88.7% for iliac crest autograft (Burkus et al., 2002). Moreover, 32% of patients in the autograft group reported donor-site discomfort at 24 months, which was avoided with INFUSE®. Similar results were demonstrated in posterolateral lumbar fusion, where INFUSE® combined with ceramic granules resulted in a fusion rate of 95% compared with 70% for autograft at 24 months (Dawson et al., 2009).

However, clinical outcomes appear to vary across orthopedic indications. In a randomized trial (NCT00853489) of critical-sized defects following open tibial fractures, INFUSE® Bone Graft combined with allograft resulted in radiographic union without secondary intervention in 58.3% (7/12) of patients at 52 weeks, compared with 81.8% (9/11) following autogenous iliac crest bone graft, although the small sample size precluded definitive conclusions regarding treatment superiority (Cannada, 2019).

The clinical performance of HEALOS® also depended on its implantation technique. HEALOS® combined with bone marrow aspirate (BMA) demonstrated similar results to iliac crest autograft in posterolateral lumbar fusion, whereas inferior results were observed for interbody fusion (Neen et al., 2006). In another study, the fusion rate after HEALOS®/BMA implantation was lower compared with local bone graft both at 2 years (29.6% vs. 63.0%) and 3 years (36.8% vs. 78.9%) (Kunakornsawat et al., 2013). Conversely, HEALOS®/BMA used in combination with interbody cages and posterior fixation resulted in fusion in 95% of treated levels (Carter et al., 2009). However, this study lacked a control group, and the additional mechanical fixation limited the evaluation of the HEALOS® contribution to bone regeneration.

NOVOCART® 3D has been clinically evaluated for matrix-associated autologous chondrocyte transplantation in focal cartilage defects of the knee. In an initial case series, 23 patients evaluated at 24 months demonstrated significant improvements in most clinical outcome measures, accompanied by a mean MOCART score of 73.2 ± 12.4 (Lukas et al., 2014). Similar improvements in clinical and radiological outcomes have been reported in subsequent studies, including follow-up extending to 5–7 years (Eichinger et al., 2024). More recently, a prospective multicenter study of 80 patients demonstrated a mean improvement in subjective IKDC score of 30.5 ± 21.5 points at 36 months, with surgery- or product-related adverse events reported in 12.5% of patients (Lenz et al., 2025).

## 6 Injectables

Injectable collagen-based medical devices comprise several formulations that differ in their physical form and clinical function. In this review, they include liquid injectable formulations, such as dermal fillers and collagen solutions used in orthopedics, as well as more viscous gels or pastes used for filling bone defects or fistula tracts.

### 6.1 Dermatology

Collagen fillers are increasingly used in cosmetology and dermatology due to their ability to restore skin structure with minimal rehabilitation time, while minimizing bruising and injection pain (Bauman et al., 2004). With age, the production and content of collagen in the skin decreases, which has stimulated the development of commercial collagen-based products to restore its balance (Jadach et al., 2024).

Сollagen fillers should have high water retention capacity, adhesiveness and mechanical stability (Salvatore et al., 2023). The optimal degradation time of collagen fillers for clinical applications has been determined to be more than 12 months, while some studies have achieved stability of collagen fillers for more than 18 months by using a novel ribose-based cross-linking technique (Monstrey et al., 2007). Biological and mechanical stability can be improved not only by strong cross-linking of collagen, but also by using fibrillar forms that are more resistant to degradation by metalloproteinases in the organism (PITARU et al., 2007).

Another important feature of fillers is their low immunogenicity. Early bovine collagen fillers were associated with a risk of hypersensitivity reactions and therefore required pre-treatment skin testing. This requirement, together with the relatively short duration of the clinical effect and the increasing availability of hyaluronic acid fillers, contributed to a decline in their use and eventual discontinuation from the U.S. market (Yang et al., 2024). Consequently, porcine collagen, which is considered less immunogenic, has also gained popularity in fillers over the past decade (Struck, 2008).

#### 6.1.1 Product information

For the production of collagen fillers, the manufacturers usually use the sources of bovine origin: Collost® (7% or 15% wt.), Zyplast® (3.5% wt), BellaFill® (3.5% wt.). However, there are fillers of porcine origin: Evolence® (3.5% wt.), Sunmax FULLSGEN® (3.5% wt.) (Supplementary Table S3a). The main raw material processing technology is the pepsin digestion (Zyplast®, Evolence®), but an alkaline-salt treatment method can be also applied (Collost®). To achieve longer biodegradation timeframes, the manufacturers use the chemical cross-linking of collagen with glutaraldehyde (Cosmoplast®, Zyplast®, Sunmax FULLSGEN®) (Peng et al., 2017) or d-ribose Evolence® (GLYMATRIX® technology) (DRAELOS, 2010). In addition to glutaraldehyde cross-linking, the FULLSGEN® filler also utilizes cross-linking with epoxy compounds and carbodiimide, which creates a triple mechanism for collagen stabilization and contributes to its maximum resistance to biodegradation after injection (Figure 5).

**FIGURE 5:**
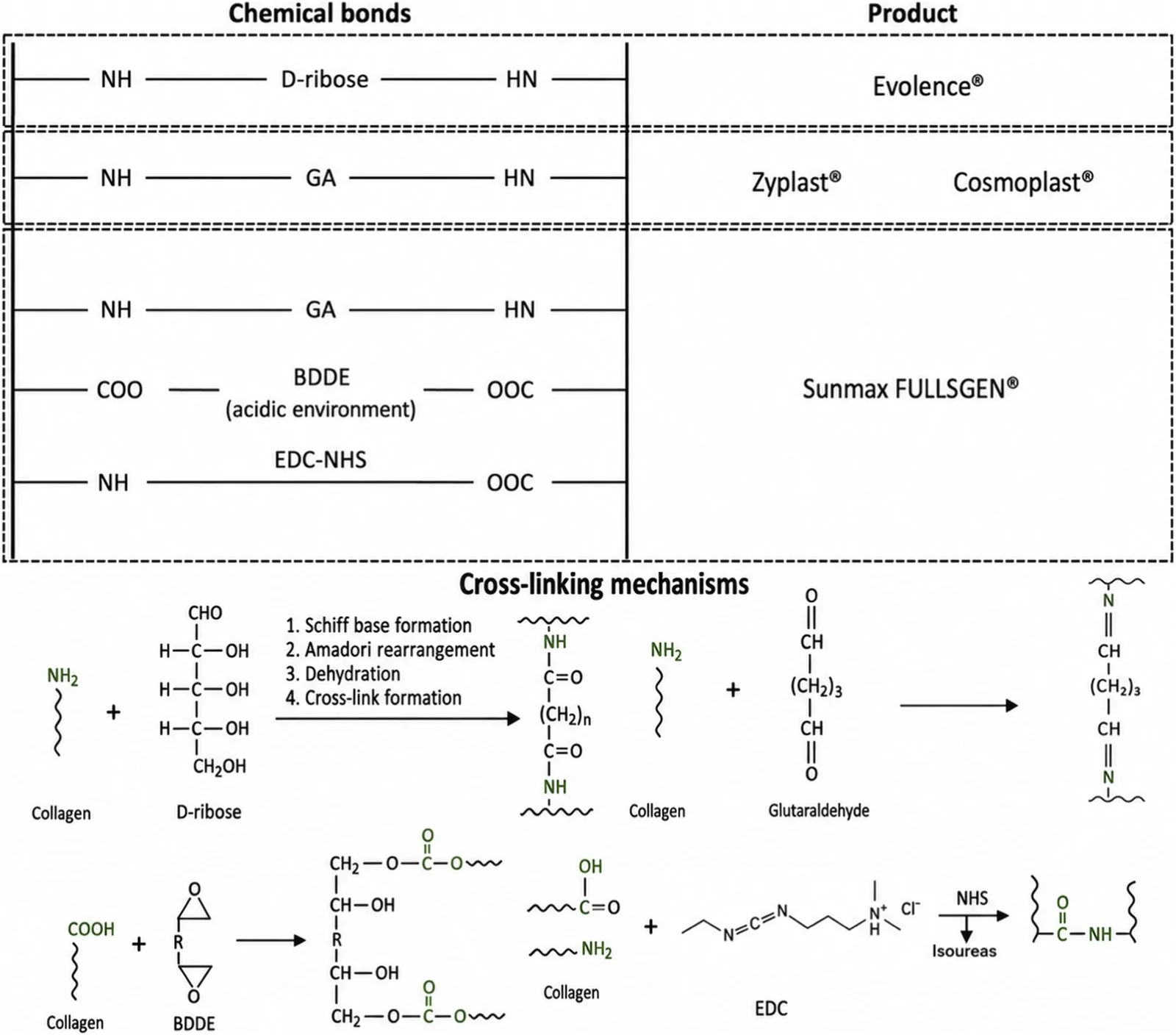
Cross-linking chemistries used to stabilize commercial collagen-based biomaterials. Upper panel: commercially available collagen products classified according to chemical cross-linking type. Lower panel: schematic representation of the corresponding chemical reactions. Abbreviations: GA - Glutaraldehyde, EDC-NHS -1-ethyl-3-(3-dimethylaminopropyl) carbodiimide/N-hydroxysuccinimide, BDDE - 1,4-Butanediol diglycidyl ether.

Another strategy for longer biodegradation timeframes is the introduction of polymethylmethacrylate microspheres to filler composition (BellaFill®): with time they become encapsulated and thus allow for long-lasting augmentation effect. As well, to achieve the reduction of the local pain under injection, the lidocaine can be added to the composition (Zyplast®, Cosmoplast®, BellaFill®, Sunmax FULLSGEN®) (Hermanns et al., 2019). It is noteworthy that from 2010, Zyplast®, Cosmoplast®, Evolence®, and some other collagen fillers are no longer manufactured due to poor sales (San Miguel Moragas et al., 2015).

#### 6.1.2 Clinical performance

It is considered that the gold standard for the comparison among different fillers is hyaluronic acid. Hyaluronic acid (HA)-based fillers have become one of the most popular and widely used types of injectable fillers in dermatology due to their excellent safety profile, efficacy, versatility, and long-term biodegradation up to 2 years (Romagnoli and Belmontesi, 2008). Therefore, the characteristics of the collagen fillers considered in this review are compared to those of HA fillers where possible.

Collagen-based fillers (Collost®, Zyplast®) have demonstrated similar efficacy as hyaluronic acid injections for wrinkle correction at early follow-up period (3 weeks) (NCT03677258 (Manturova et al., 2018); NCT00876265 (Narins et al., 2010a)). However, later (24 weeks), HA fillers showed better results compared to collagen injection (Zyplast®): the global aesthetic improvement scale scores were 2.37 ± 0.77 and 1.85 ± 0.86, the wrinkle severity rating scale scores were 1.08 ± 0.74 and 0.50 ± 0.73 for the HA filler and Zyplast® groups, respectively. It is worth noting that the therapy efficacy scores in the Zyplast® group decreased faster and to a greater extent than in the HA filler group (Narins et al., 2010b).

Collagen filler (Cosmoplast®) was also compared with polylactide-based filler (NCT00444210: comparative study (Narins et al., 2010b); NCT00444353: long-term follow-up (Brown et al., 2011)) for the correction of moderate to severe nasolabial wrinkles. As in the case of comparison with HA-based fillers, the collagen filler showed improvements in skin recovery rates only at short-term follow-up (3 months), while the polylactide-based filler-maintained improvements up to 25 months of follow-up.

The optimization of filler cross-linking technology can improve its long-term stability. For example, sugar cross-linked Evolence® demonstrated better long-term stability in maintaining clinical wrinkle correction and resistance to biodegradation compared to Zyplast® at the 18-month follow-up in 9 out of 11 patients (Monstrey et al., 2007). Another approach is a combination of collagen cross-linking mechanisms (Sunmax FULLSGEN®). In the study NCT03844529, the FULLSGEN® filler was compared with a previous-generation filler (FACIALGAIN®), which was less resistant to biodegradation due to a lower number of collagen cross-links (Yang et al., 2024). Efficacy was assessed using the Wrinkle Severity Rating Scale (WSRS) (a 5-point wrinkle severity scale). After 13 months, the reduction in wrinkle severity was 3.3–6.1 times greater in the FULLSGEN® group compared to the FACIALGAIN® group.

Of note, the enhanced cross-linking in FULLSGEN® did not lead to an increase in the number of immunological complications in a study by Tai et al. (Tai et al., 2024): skin prick tests were negative in all patients (n = 124), no significant changes in immunoglobulin levels were detected, and the only moderate allergic reaction following treatment was observed in just 0.81% of patients.

The clinical trial of the BellaFill® filler (NCT03327311) showed that the polymethyl methacrylate spheres in filler formulation promoted the synthesis of proteoglycans and type III collagen at the histological level starting in the first week, and triggered the synthesis of procollagen I starting in the fourth week, indicating the formation of more mature dermal collagen (Ronan et al., 2019). However, this study confirms the mechanism of biostimulation rather than clinical efficacy, as it included only 11 participants and lacked a control group.

In the NCT02642627 study, BellaFill® was used to treat atrophic post-acne scars. After 7 months, the response rate was 95%, with a reduction in the mean Acne Scar Assessment Scale (ASAS) score (a 5-point scale for scar severity) from 3.33 to 2.2 points (Joseph et al., 2019). In the comparative study NCT02643628, for the treatment of atrophic post-acne scars, the addition of BellaFill® to microneedling resulted in a statistically significant but moderate reduction in ASAS severity at 24 weeks compared to microneedling alone: 3.00 ± 0.91 versus 3.71 ± 0.75, respectively. However, no data were provided for the control group at the 36-week mark, which limits conclusions regarding the long-term benefits of this combined therapeutic approach (Biesman et al., 2019).

Thus, despite good performance in the first months after injection, the value of collagen fillers remains questionable due to faster degradation at later time points compared with hyaluronic acid fillers (from 3–6–18 months) (Witmanowski and Błochowiak, 2020). Moreover, collagen dermal fillers may not provide as significant volumizing effect as HA fillers for deep wrinkles or areas requiring more volume, since HA naturally attracts more water swelling by thousands of percents (Lee et al., 2015).

Nevertheless, there is growing renewed interest in collagen fillers in aesthetic medicine and dermatology as stimulators of endogenous extracellular matrix regeneration, while traditional inert hyaluronic acid fillers are being reevaluated (Haddad et al., 2022). The main advantage of hyaluronic acid, its strong hydrating effect, is also its drawback: after injections, swelling, product migration, and hardening of the surrounding tissues may occur (Baranska-Rybak et al., 2024). In this case, collagen fillers or collagen as an additive to HA-based fillers can be considered a component capable of improving skin quality by activating reparative processes, rather than merely “mechanically” filling wrinkles.

One such example is the Karisma® formulation, which combines high-molecular-weight non-cross-linked hyaluronic acid with recombinant human type I collagen. The NCT06152718 study evaluated the efficacy of Karisma in patients with signs of skin aging and post-acne scars. After 2 months, the authors observed a statistically significant reduction in wrinkle depth and skin roughness. However, since the study lacked a control group, it does not prove Karisma® superiority over conventional fillers (Di Rosa et al., 2024). In addition, a study NCT06336044 has been registered to evaluate Karisma for the treatment of wrinkles, scars, and signs of sagging skin. However, at this time, this is a pre-marketing trial with a “recruiting” status and no published results. Therefore, it can only be used as evidence of ongoing clinical interest in hybrid fillers based on hyaluronic acid and collagen.

### 6.2 Dentistry

Injectable forms of collagen are used in dentistry to prevent bone volume loss after tooth extraction or inflammatory diseases. Gel-like formulations of collagen containing bone granules have advantages over non-hydrated bone granule forms by being a “ready-to-use” product, which reduces preparation time during surgery and reduces the risks of microbial contamination (Falacho et al., 2021). The high adhesiveness of these formulations facilitates application, providing stability of the material at the defect site, which is effective for horizontal bone augmentation and preservation of the socket (Xie et al., 2024; Attia, 2017). Viscous collagen base also promotes osteogenesis by providing a three-dimensional framework for the growth and differentiation of osteoblasts and stimulates the formation of new blood vessels (Matthews et al., 2014; Nguyen et al., 2017).

#### 6.2.1 Product information

There are two OsteoBiol® products made by Tecnoss on the market (MP3 and Gel 40) (Supplementary Table S3b). Both gels are based on atelocollagen types I and III of animal origin with a difference in concentration: 10% wt. and 40% wt., respectively. In addition, the products contain a bone granular mixture, but the particle size is different: 600–1000 or 1000–2000 μm for MP3 and <300 μm for Gel40 (Tecnoss Dental Srl, n.d.; Pagliani et al., 2012).

#### 6.2.2 Clinical performance

The studies showed the same efficacy of collagen-based gels with added bone granules (Gel 40 and MP3) and dry collagenous cortico-cancellous bone graft Gen-Os® in different clinical situations over 12 months. In the case of isolated intraosseous periodontal defect treatment, the extent of pocket depth reduction was similar for Gel40 (3.5 ± 1.0 mm) and Gen-Os® (3.9 ± 1.7 mm) (NCT04782921 (Kobe et al., 2024)). As well, in alveolar crest and maxillary sinus floor augmentation, the defect mineralization was not significantly different between MP3 and Gen-Os®: 52.2% ± 15.0% and 61.4% ± 16.3% respectively (Pagliani et al., 2012). The MP3 product was also compared to cortical bone material (Apatos®) for preserving the socket after tooth extraction (NCT02644070 (Marconcini et al., 2018)). It was shown that the average marginal bone loss after 4 years of follow-up was similar for both products.

Clinical studies showed that injectable collagen gels have no significant advantages over non-hydrated bone granules, although they may be easier to use by the physician due to the convenient filling of bone defects. Prospectively, the addition of growth factors, such as bone morphogenetic proteins, may improve the efficacy of these forms by promoting new bone growth through prolonged release from the gel forms (Gillman and Jayasuriya, 2021; McKay et al., 2007).

### 6.3 Coloproctology

The introduction of hydrated collagen forms (pastes) has been proposed as an approach to treat complex anal fistulae (Bayrak and Altıntas, 2018). The conventional surgical methods of fistula treatment can cause up to 50% of continence issues postoperatively (Darwish et al., 2023; Aly, 2014), while the existing minimally invasive methods have not shown satisfactory outcomes (El-Gazzaz et al., 2010; Safar et al., 2009; Gisbertz et al., 2005; Singer et al., 2005). The aim of developing collagen pastes is to achieve more satisfactory clinical results due to the natural pro-regenerative ability of collagen and its long-term stability (Gu et al., 2019). The advantage of the paste form is also its pliability and ability to easily fill the fistula volume (Giordano et al., 2018).

#### 6.3.1 Product information

The example of collagen paste for fistula treatment is Permacol® (Supplementary Table S3c). It has high collagen content and is made of the decellularized porcine dermis that is purified from other substances. The extracted collagen matrix is cross-linked, homogenized, and suspended in saline to obtain a ready-to-use injectable paste for direct filling of fistula tracts (Brunner et al., 2019; Giordano et al., 2018).

#### 6.3.2 Clinical performance

In the clinical trial NCT01624350 (Giordano et al., 2018), Permacol® demonstrated high efficacy in treating anorectal fistulas, achieving fistula closure in 56.7% of patients after 6 months and a recurrence rate of 29% after 12 months. The incidence of serious complications was low (4%). Other studies have also confirmed the efficacy of Permacol® and reported no adverse effects on sphincter function (Fabiani et al., 2017). However, these studies lacked control groups for comparison. In a controlled study (NCT01528995 (Rydningen et al., 2017)) on the treatment of fecal incontinence after anal sphincter injury, Permacol® showed statistically worse results compared to sacral neuromodulation regarding quality-of-life index, fecal and urinary continence. Further trials, including comparisons with other treatment methods such as fibrin glue, seton placement, and the ligation of the intersphincteric fistula tract (Hong et al., 2014), are needed to form a definitive opinion on the efficacy of Permacol®.

### 6.4 Traumatology and orthopedics

#### 6.4.1 Tendinopathies

Collagen injections are utilized to treat tendinopathies, in particular, rotator cuff and Achilles tendon disorders (Millar et al., 2021). Current conservative treatment methods are limited by incomplete recovery or significant side effects (Shim et al., 2023). While surgical interventions are effective, they are associated with high risks and require a lengthy recovery period (Schmucker et al., 2020). Studies have shown that injecting collagen into damaged tendons can improve collagen metabolism and slow down degenerative changes in connective tissue, enhancing its mechanical properties and structure, and restoring functionality (Godek et al., 2022; Randelli et al., 2018; de Girolamo et al., 2019.)

##### 6.4.1.1 Product information

The manufacturer Guna produces numerous collagen-based injectable formulations, among which are MD-Tissue Collagen® and MD-Shoulder Collagen®. Together with RegenSeal® (Cellontech), these products are used for the treatment of tendinopathies (Supplementary Table S3d). All of them are the injections based on type I porcine collagen. Of note, MD-Tissue Collagen® and MD-Shoulder Collagen® are based on hydrolyzed collagen form.

As stated by manufacturer, MD-Tissue Collagen® contains ascorbic acid, pyridoxine hydrochloride, riboflavin, and thiamine hydrochloride. These substances possess antioxidant properties and support normal nervous system function (Rivero et al., 2024; FEEDAP EP on A and, 2010; Mrowicka et al., 2023; Olfat et al., 2022; Bechara et al., 2022). MD-Shoulder Collagen® includes an extract from the iris plant, which also exhibits antioxidant activity (Khatib et al., 2022).

##### 6.4.1.2 Clinical performance

Collagen-based injectables have been applied for reducing pain and improving functional activity in the most common tendon pathologies of the upper and lower extremities, such as the rotator cuff (NCT05489484, MD-Shoulder Collagen®) and the Achilles tendon (NCT05464498, MD-Tissue Collagen®). However, the results of these studies have not yet been published.

In a study with published results (NCT04492748 (Godek et al., 2022)), the effectiveness of MD-Shoulder Collagen®, platelet-rich plasma (PRP), and their combination was compared for the treatment of rotator cuff partial tears. After 6 months, no significant differences were found in pain reduction and improvement of functionality among groups, indicating equal efficacy of collagen injections and PRP. However, the choice of PRP as a comparison group is questionable, since PRP is not considered a first-line treatment for tendinopathies, and moreover, in some studies, its efficacy even was not proved (Franchini et al., 2018; Nauwelaers et al., 2021; Liu et al., 2019b).

Collagen injection does not always lead to structural tissue repair, despite the clinical benefit, as demonstrated in a study by Kim et al. (Kim et al., 2019). RegenSeal® was administered during arthroscopic repair of a full-thickness rotator cuff tear and compared with a group in which repair was performed without collagen injection. Although RegenSeal® reduced pain in patients by nearly 2–3 times on the visual analogue scale (VAS) in the short term (up to 2 weeks), the difference between the groups had clinically resolved by 6 months. According to MRI data at 6 months, the rate of recurrent rotator cuff tears also did not decrease: 11.5% in the RegenSeal® group vs. 6.7% in the control group.

#### 6.4.2 Cartilage repair

Collagen injectables are used to treat cartilage defects caused by osteoarthritis, the most common degenerative musculoskeletal disease for which mainstream therapies are associated with a number of complications after long-term use (Tarantino et al., 2023; Loeser et al., 2016; Hunter, 2011). Therefore, intra-articular collagen injections have become popular as a minimally invasive and safe way to regenerate cartilage. Collagen can be injected into the joint as a solution with intact or partially hydrolyzed molecular structure (De Luca et al., 2019) or in polymerized form to coat the surface of cartilage in microfractures, promoting its repair (D’Antimo et al., 2017).

The mechanisms by which injectable collagen formulations exert their effects on articular cartilage remain incompletely elucidated. Experimental studies suggest that polymerized collagen may promote chondrocyte and synovial cell proliferation, enhance the synthesis of extracellular matrix proteins, and modulate inflammatory responses by reducing the release of pro-inflammatory mediators (Interleukin-1 beta and Tumor necrosis factor-α) while increasing the production of the anti-inflammatory cytokine Interleukin-10 (Furuzawa-Carballeda et al., 2009a; Furuzawa-Carballeda et al., 2009b). In contrast, investigations of hydrolyzed collagen indicate that this form does not significantly affect the expression of matrix metalloproteinases or their endogenous inhibitors, but may stimulate type II collagen synthesis and suppress type I collagen deposition (De Luca et al., 2019).

##### 6.4.2.1 Product information

The products are primarily based on porcine tissues (CartiFill®, CartiZol®, MD Tissue Collagen®, MD Knee®) (Supplementary Table S3d). The predominant production technology involves pepsin treatment of the raw material followed by dissolution in a buffer solution (CartiFill®, CartiZol®).

The main difference between CartiFill® and CartiZol® is their application: CartiFill® serves as a support matrix for cartilage repair procedures, whereas CartiZol® is used as an intra-articular collagen injection for pain relief and functional improvement. Alternative manufacturing approaches also exist. For example, BioCartilage® is produced through gentle hypothermic dehydration and milling of allogeneic human cartilage (Commins et al., 2021).

Additional components are added to collagen base for specific properties. For example, polyvinylpyrrolidone (PVP), used in Fibroquel®, possesses complex-forming properties and improves tissue adhesion, while collagen-PVP complexes have been reported to exert anti-inflammatory and anti-fibrotic effects (Cervantes-Sánchez et al., 2003; Li et al., 2016). Plant-derived extracts, such as Arnica extract, which possess anti-inflammatory properties, are also included in certain formulations (MD-Knee®) (Sugier et al., 2023). In addition, some collagen fillers are recommended for use in combination with fibrin glue to promote polymerization and fixation at the defect site (CartiFill®).

##### 6.4.2.2 Clinical performance

CartiFill® has been successfully used for cartilage regeneration in the treatment of osteochondral lesions of the talus (NCT02519881 (Lee et al., 2020)). The MRI-based cartilage regeneration score in the CartiFill® + microfracture group was 64.49 ± 18.27, compared with 53.01 ± 12.14 in the control group (microfracture alone). CartiFill® has also demonstrated efficacy in the treatment of osteochondral lesions of the knee joint in NCT02539030. CartiFill® was used as an adjunct to microfracture therapy for knee cartilage defects and was compared with the results of microfracture therapy alone. In the CartiFill® group, a higher rate of clinically significant pain reduction was observed after 1 year (77.8% vs. 52.3%), while the probability of achieving 50% or greater defect filling as determined by MRI after 12 months was 3.984 times higher (Kim et al., 2020).

BioCartilage® was clinically applied as the adjunct to microfracture procedure in the treatment of knee joint cartilage defects (NCT02203071). Such combination, according to magnetic resonance imaging (MRI) data, resulted in higher rates of cartilage regeneration compared to traditional isolated bone marrow stimulation. After 2 years of follow-up, the patients in Biocartilage® group showed higher functional recovery scores (Knee Injury and Osteoarthritis Outcome Score of 85.9 ± 12 vs. 71.6 ± 18 in the control group), as well as physical activity score (Marx Activity Rating Scale) [9.5 ± 5 vs. 3.4 ± 4.2 in the control group].

A hydrolyzed collagen-based injectable (MD-Knee®) has also been investigated for the treatment of knee osteoarthritis. Its clinical efficacy was reported to be comparable to that of intra-articular sodium hyaluronate injections, which are widely regarded as the current standard of care. In addition, it was reported that MD-Knee® can be a potentially more cost-effective therapeutic alternative (Martin Martin et al., 2016).

The injectable product CartiZol® was used in the NCT02539095 to treat patients with chondromalacia, osteoarthritis, or traumatic arthritis, while a placebo group receiving an injection of saline served as the control. After 6 months, the proportion of patients reporting improvement was 71.3% in the CartiZol® group and 57.6% in the placebo group. According to physicians’ assessments, improvement was observed in 72.3% and 61.6% of patients, respectively. However, these data primarily confirm a symptomatic (clinical) effect, and no MRI assessment of cartilage tissue regeneration was performed.

Additionally, in the NCT07511777 CartiZol® was compared with intra-articular hyaluronic acid injections in patients with knee osteoarthritis. However, the results of this study have not yet been published. Nevertheless, in a retrospective study using a similar protocol, intra-articular injections of CartiZol® demonstrated clinical efficacy at least equivalent to that of hyaluronic acid injections in terms of pain reduction and improvement in functional outcomes in patients with knee osteoarthritis (In et al., 2025).

## 7 Limitations

In this review, a relatively narrow search strategy was employed, without extensive use of synonyms for the term “device” (e.g., implant, matrix, scaffold). It should also be noted that, in some databases, descriptions of certain collagen-based products do not explicitly include the term “collagen”, and instead, it refers only to proprietary trade names. Eventually, it may have limited the overall completeness of our analysis.

In addition, clinical trial retrieval was restricted to ClinicalTrials.gov and did not include other major international trial registries, such as the WHO International Clinical Trials Registry Platform, the EU Clinical Trials Register (EudraCT/CTIS), or the Chinese Clinical Trial Registry. Consequently, our analysis may under-represent products that are primarily tested and marketed in the European Union, China, or other regions with independent registration systems. We acknowledge that an analysis focused on clinical trials may be of limited utility for investigators aiming to initiate frontier or disruptive research, as insights for the development of such technologies are often derived from emerging approaches that are typically explored in preclinical studies.

On the other hand, the transition from preclinical research to clinical application is frequently associated with high failure rates (Fosse et al., 2023). Consequently, initiating device development solely on the basis of preclinical evidence may pose substantial challenges, as researchers must contend with a large volume of ineffective or non-translatable knowledge. Accordingly, to establish a robust and rational development strategy, it is advantageous to first conduct a comprehensive assessment of the existing clinical trial landscape, as the initiation of clinical studies is generally driven by demonstrated clinical need, economic feasibility, and technological maturity.

## 8 Conclusions and future perspectives

We systematized data on collagen-based medical devices by comparing clinical outcomes with the technical requirements that an “ideal” implant for a specific indication must meet. In particular, the requirements for the reviewed devices concerned not only the initial properties of the raw materials but also the conditions and the duration during which the intended function of the medical device must be maintained.

The identified limitations of collagen-based devices can be grouped into three priority areas for further development. In cases where premature degradation compromises the sealing properties or leads to volume loss, the key challenge is to further stabilize the collagen matrix. If a biologically stable matrix provides insufficient guidance for tissue regeneration or mechanical support, changes to its architecture or composition are required. If inflammatory reactions or foreign-body reactions hinder integration with tissues, the conditions for purification and processing of the product must be revised. The proposed technological approaches to product modification may overlap, and improving one property may simultaneously compromise another. For example, an increase in resistance to biodegradation in hemostatic sponges should be evaluated considering their blood absorption capacity, and in the case of implants serving as regenerative matrices, the degree of cellular infiltration must also be determined.

Table 4 shows the relationships between the limitations of each collagen-based medical device form/group and the potential perspectives for their technological improvements. Furthermore, by integrating clinical requirements, manufacturing approaches, and the relevant standards for material characterization and biological evaluation, this review establishes a foundation for planning device development: from the selection of the collagen source and processing conditions to functional testing and clinical evaluation. Within this framework, the limitations observed in existing products can serve both as a guide for the design of new products and for the selection of tests needed to determine whether the proposed modifications offer an advantage over existing alternatives.

**TABLE 4:** Main limitations of representative collagen-based medical devices and potential technological directions for their improvement.

Product form	Product group/representative products	Main limitation identified	Potential technological direction for improvement
Membrane	Peripheral nerve conduits: NeuraGen®, NeuroMatrix®, NeuroFlex®	The effectiveness of hollow collagen nerve conduits decreases with increasing nerve-gap length and injury complexity, limiting their use primarily to relatively short peripheral nerve defects (Davidenko et al., 2012; Lohmeyer et al., 2009).	Development of intraluminal aligned fiber or multichannel architectures that more closely reproduce the directional organization of native nerve fascicles and provide additional guidance for axonal regeneration (Li et al., 2022b; Li et al., 2010). These architectures may be further functionalized with neurotrophic or other regenerative factors to provide sustained biochemical stimulation during nerve regeneration (Chang et al., 2017).
	Collagen/ECM matrices for rotator cuff repair with reported adverse or suboptimal tissue responses: Zimmer® Collagen Repair Patch (Permacol®), CuffPatch®	Residual cellular or xenogeneic antigenic components after incomplete decellularization and/or extensive matrix cross-linking may induce inflammatory and foreign-body responses, restrict cellular infiltration, and interfere with constructive tissue remodeling (Delgado et al., 2015; Kasravi et al., 2023).	Development of next-generation decellularization technologies, including reagent-free electric-field processing, deep eutectic solvent-based decellularization, and advanced supercritical-fluid systems, aimed at accelerating cell removal while minimizing chemical residues and preserving native ECM architecture and bioactivity (Ren et al., 2025).
Sponge	Mineral-containing collagen matrices for dental bone regeneration: Bio-Oss® Collagen, NuOss XC®	Slow resorption of the mineral component may result in prolonged persistence of residual particles and incomplete replacement of the implanted material by newly formed bone (Bohner et al., 2020; Somngam et al., 2025).	Adjustment of mineral content and mineral-phase composition to better synchronize scaffold resorption with new bone formation. Potential approaches include reducing the fraction of slowly resorbing mineral and incorporating more rapidly resorbable calcium phosphates, particularly β-TCP, or developing biphasic hydroxyapatite/β-TCP compositions that combine the volume stability of hydroxyapatite with faster remodeling of β-TCP (Bohner et al., 2020; Bouler et al., 2017).
	Hemostatic/sealing collagen matrices: TachoSil®, HEMOPATCH®, Lyostypt®	Current collagen-based hemostatic matrices may be limited by formation of a superficial swollen or coagulated layer that restricts further blood penetration into the porous matrix and, depending on the hemostatic mechanism, by the time required to establish effective hemostasis (Boerman et al., 2017). In pancreatic applications, proteolytic degradation of the collagen carrier and fibrin coating can compromise seal integrity and limit protection against postoperative leakage (Adelmeijer et al., 2013; Philipp et al., 2017)	Functionalization with rapidly acting inorganic or synthetic hemostatic agents represents a potential strategy to accelerate clot formation while preserving the fluid-absorbing properties of the collagen matrix (Wang et al., 2022; Lin et al., 2023). Selection of cross-linking conditions that improve enzymatic resistance while preserving fluid uptake and porous structure (Wang et al., 2022). For pancreatic sealing, additional research directions include local inhibition of proteolysis and the use of protease-resistant recombinant collagen building blocks (Adelmeijer et al., 2013; Shen et al., 2025)
Injectable	Collagen dermal fillers: Collost®, Zyplast®, Cosmoplast®	Bovine collagen may induce hypersensitivity and immune reactions, historically necessitating pretreatment skin testing. In addition, progressive enzymatic degradation of collagen limits implant persistence and therefore the duration of the volumizing effect of temporary collagen fillers (KLIGMAN and ARMSTRONG, 1986; Keefe et al., 1992).	Reduction of source-related immunogenicity through selection of collagen sources with lower hypersensitivity potential, such as porcine or recombinant human. Improved purification, combined with advanced collagen cross-linking approaches acting through different functional groups and/or multifunctional cross-linkers strategies (Chen et al., 2025; Guo et al., 2023; Omobono et al., 2015). Development of covalently integrated collagen–hyaluronic acid hydrogels to improve volume retention relative to collagen-only formulations while supporting extracellular matrix deposition (Song et al., 2024).
	Injectable collagen-containing bone substitutes: OsteoBiol® MP3, Gel 40	Rapid resorption of the collagen-rich carrier phase may contribute to early loss of matrix volume, while the collagen component primarily improves handling and adaptation to irregular defects without necessarily providing prolonged biological stimulation of bone regeneration (Saleh et al., 2025).	Development of cross-linked injectable collagen matrices combining defect-adaptable delivery with prolonged matrix persistence and sustained local delivery of osteogenic or other bioactive agents. Incorporation of growth factors or other osteoregenerative molecules into the stabilized collagen hydrogel network could provide controlled release and maintain biological stimulation during the early stages of bone regeneration (Kou et al., 2026).

## Glossary

ASAS: Acne Scar Assessment Scale
ASES: American Shoulder and Elbow Surgeons questionnaire
ACL: anterior cruciate ligament
BMA: bone marrow aspirate
CAF: coronally advanced flap
CSF: cerebrospinal fluid
CXL: corneal cross-linking
dsDNA: double-stranded deoxyribonucleic acid
ECM: extracellular matrix
EDC: 1-ethyl-3-(3-dimethylaminopropyl)carbodiimide
EDC–NHS: 1-ethyl-3-(3-dimethylaminopropyl)carbodiimide/N-hydroxysuccinimide
EMD: enamel matrix derivative
FDBA: freeze-dried bone allograft
FDA: Food and Drug Administration
FGF: fibroblast growth factor
GBR: guided bone regeneration
GLP: Good Laboratory Practice
HA: hyaluronic acid
IKDC: International Knee Documentation Committee
ICL: implantable collamer lens.
IOL: intraocular lens
IOP: intraocular pressure
MACT: matrix-associated autologous chondrocyte transplantation
MMC: mitomycin C
MOCART: magnetic resonance observation of cartilage repair tissue
MRI: magnetic resonance imaging
NCT: ClinicalTrials.gov trial identifier
PEG–NHS: N-hydroxysuccinimide-functionalized polyethylene glycol
PDGF: platelet-derived growth factor
PEG: polyethylene glycol
PLGA: poly(lactic-co-glycolic acid)
POPF: postoperative pancreatic fistula
PRP: platelet-rich plasma
PTFE: polytetrafluoroethylene
PVP: polyvinylpyrrolidone
rhBMP-2/7: recombinant human bone morphogenetic protein-2/7
SEM: scanning electron microscopy
s2PD: static two-point discrimination
**scCO**_**2**_: supercritical carbon dioxide
SIS: small intestinal submucosa
TGF-β: transforming growth factor beta
TNMD: tenomodulin
UV: ultraviolet
VAS: visual analogue scale
WSRS: Wrinkle Severity Rating Scale
β-TCP: β-tricalcium phosphate
