# Supplementary material for: Collagen-based medical devices: technological basis and clinical studies
Source: Front Bioeng Biotechnol. 2026 Oct 29;14:1971640. doi: 10.3389/fbioe.2026.1971640 (PMC13754717; doi:10.3389/fbioe.2026.1971640)
Supplement: Supplementary file 1 [file Table1.docx]

SUPPLEMENTARY MATERIALS

| **Product / manufacturer** | **Source and type** | **Tissue / organ** | **Manufacturing technologies** | **Related patents** | **Clinical trials** |
| --- | --- | --- | --- | --- | --- |
| **a) Neurosurgery** | | | | | |
| AxoGuard® Nerve Protector/ AxoGuard® Nerve Connector, Cook Biotech | Porcine | SIS | **Processing technology:** Decellularization; vacuum pressing (4 layers)  construct  **Sterilization:** Ethylene oxide | US6206931;  US6241981;  US7652077;  US6358284 | NCT03941327;  [128] |
| NeuraGen® Nerve Guide/ 3D Nerve Guide Matrix, Integra LifeSciences | Bovine, type I | Tendon | **Extraction technology:** Enzymatic and alkali treatment  **Processing technology**: Freeze-drying  **Supplements:** Uniaxially-aligned porous collagen matrix with chondroitin-6-sulfate (NeuraGen® 3D Nerve Guide Matrix)  **Cross-linking**: Chemical tanning agents | US6969523B1;  EP2255833A2;  US5019087A | NCT05339594;  [128] |
| Avance®, Axogen Corporation | Human (allogeneic) | Peripheral nerve | **Processing technology**: Decellularization  **Storage medium:** Lactated Ringer’s Solution. | US 9572911B2 | NCT00948025 [130] |

| NeuroMatrix®/ NeuroFlex®, Collagen Matrix | Bovine, type I | Tendon | **Extraction technology**: Acid–salt and Alkali–salt  **Processing technology**: Freeze-drying  **Cross-linking**: Formaldehyde (vapor) | US6716225B2 | NCT00948025 [130] |
| --- | --- | --- | --- | --- | --- |
| Remplir™, Orthocell | Porcine, type I | Peritoneum | **Processing technology:** Decellularization, SMRT™; air-drying; mechanical stretching | US10888638B2 | [129] |
| **(b) Dentistry** | | | | | |
| Ez Cure®, Biomatlante SA | Porcine, type I/III | Skin | **Cross-linking**: Formaldehyde | - | [148] |
| Creos™ Xenoprotect, Matricel GmbH | Porcine | - | **Processing technology**: Decellularization; dehydration and air-drying  **Supplements:** Elastin | US20040265785A1 | NCT02373787;  [147] |
| OSSIX® Volumax, Datum Dental | Porcine, type I | Tendon | **Processing technology:** Fiber reconstitution; compression; multi-layer scaffold formation; freeze-drying  **Cross-linking**: Sugar cross-linking, GLYMATRIX® | US20240016977A1 | NCT03457298 [152] |
| Mucograft®, Geistlich Pharma | Porcine, type I/III | - | **Processing technology**: Vacuum drying; freeze-drying; combination of compact and spongy layers | EP1252903B1;  WO 99/19005;  WO 93/11723; US5837278A | NCT03551145 [158];  NCT00902876 [156];  NCT01808794 [143];  NCT02456337 [155];  NCT02697890 [144] |
| Bio-Gide®, Geistlich Pharma | Porcine, type I/III | - | **Processing technology**: Decellularization; degreasing; dehydration | US5837278A | NCT02373787 [147];  NCT03457298 [152];  [145,146] |
| Vitala®, Osteogenics Biomedical | Porcine | Pericardium | **Processing technology**: Decellularization; freeze-drying; air/vacuum drying  **Cross-linking**: UV | US12472281B2 | NCT04257097 [149,150] |
| Jason membrane®, botiss biomaterials | Porcine, type I/III | Pericardium | **Processing technology**: Decellularization; freeze-drying | - | [145,146] |
| Gintuit™, Organogenesis | Bovine, type I |  | **Processing technology:** Living bilayer cellular sheet formation (keratinocytes+fibroblasts) | US5536656A | NCT00587834 [159] |
| DynaMatrix®, Cook Biotech | Porcine | SIS | **Processing technology:** Decellularization; compression (4 layers); freeze-drying | US6206931B1 | NCT01808794 [143] |
| PriMatrix® Dermal Repair Scaffold, Integra LifeSciences | Bovine | Skin | **Processing technology**: Decellularization; air/freeze-drying | US6696074B2 | [157] |
| Striate+™, Orthocell | Porcine, type I | Peritoneum | **Processing technology:** Decellularization, SMRT™; air-drying; mechanical stretching | US10888638B2 | ACTRN12615000027516 [67];  [151] |
| **(c) Ophthalmology** | | | | | |
| ABCcolla®, ACRO Biomedical | Porcine | Cornea | **Processing technology:** Decellularization | US10660988B2 | NCT04054817 |
| LinkCor®, LinkoCare Life Sciences | Porcine, type I | Skin | **Processing technology: Vacuum evaporation, molding**  **Cross-linking**: EDC-NHS, UV-riboflavin | US10568987B2 | NCT04653922 [24] |
| Visian® ICL, STAAR Surgical Company | Porcine, type IV | Eye | **Extraction technology:** Alkali treatment  **Supplements**: HEMA, MHBPH  **Cross-linking**: Radiation | US5910537A | NCT02833649 [173] |
| **XENIA® Corneal Implant / Gebauer™ Lenticule**, Gebauer Medizintechnik | Porcine | Cornea | **Processing technology**: Decellularization **Processing technology**: Compression **Cross-linking**: UV | US12350144B2;  WO2021130276A1 | NCT04741230 |
| **(d) Orthopedics** | | | | | |
| REGENETEN®, Smith & Nephew | Bovine, type I | Tendon | **Processing technology**: Fiber deposition; layer stacking; freeze-drying | US20240374369A1;  US20220401627A1 | NCT04444076 [182];  NCT04450342;  NCT06857084 [410];  [180,181] |
| **CuffPatch™,** Organogenesis | Porcine | SIS | **Processing technology**: Decellularization; multi-layer matrix formation **Cross-linking:** EDC | US6599690B1 | - |
| **Restore®,** DePuy Orthopaedics | Porcine | SIS | **Processing technology**: Decellularization; multi-layer matrix formation | - | NCT00208338;  [192–196] |
| GraftJacket®, LifeCell | Human | Skin | **Processing technology**: Decellularization | US5336616A | NCT01987973 [183];  **NCT04975581 [185];**  [184] |
| ArthroFLEX®, LifeNet Health | Human | Skin | **Processing technology**: Matracell® decellularization  **Storage:** Preservon® technology | US6734018B2;  US6743574B1;  US7338757B2 | NCT06215417;  NCT03511547;  NCT03551509;  [184] |
| Conexa™, LifeCell Corporation | Porcine | Skin | **Processing technology**: Decellularization | **US8735054B1;**  US9150318B1;  US10307510B2;  US8469779B1 | **NCT01025037 [189];**  **[190,191]** |
| TissueMend®, TEI Biosciences | Bovine, type I/III | Skin | **Processing technology**: Decellularization; air/ freeze-drying | US6696074B2;  US7354702B2;  US9011895B2 | - |
| Zimmer® Collagen Repair Patch, Tissue Science Laboratories | Porcine | Skin | **Extraction technology**: Decellularization **Cross-linking:** Hexamethylene diisocyanate | US5397353A | [187,188] |
| OrthADAPT®, Pegasus Biologics | Equine, type I | Pericardium | **Extraction technology**: Decellularization **Cross-linking:** EDC | US9694037B2 | Eudra-CT 2007-007630-19 [197] |
| CelGro ™, Orthocell | Porcine, type I | Peritoneum | **Processing technology:** Decellularization, SMRT™; air-drying; mechanical stretching | US10888638B2 | [179] |

**Table S1.** Membrane-form collagen-based medical devices.

| **Product / manufacturer** | **Source and type** | **Tissue / organ** | **Manufacturing technologies** | | **Related patents** | | **Clinical trials** | |
| --- | --- | --- | --- | --- | --- | --- | --- | --- |
| **(a) Dentistry** | | | | | | | | |
| Bio-Oss Collagen®, Geistlich Pharma | Porcine | Connective tissue | **Processing technology**: Freeze-drying  **Supplements**: Bone granules | | US5573771;  US5167961 | | NCT03422458 [204];  NCT03395145 [205];  NCT03659617 [206];  NCT04338516 [209] | |
| Ossix Bone®, Dentsply Sirona | Porcine, type I | Tendon | **Extraction technology**: Pepsinization  **Processing technology**: Freeze-drying  **Supplements:** Hydroxyapatite  **Cross-linking**: Sugar cross-linking, GLYMATRIX® | | US20080026032A1;  US6682760B2;  US6346515B1 | | NCT04338516 [209] | |
| Hemocollagene®, Septodont | Bovine, type I | - | **Processing technology**: Freeze-drying | | - | | NCT05171231 | |
| NuOss XC®, Collagen Matrix | Bovine, type I | - | **Processing technology**: Freeze-drying, compressing  **Supplements**: bone granules | | US8877220B2 | | NCT01391767 | |
| OsteoGen Plug®, Impladent | Bovine, type I | Tendon | **Supplements**: Calcium apatite | | US9238047B1 | | NCT05419778 [210] | |
| **(b) Ophthalmology** | | | | | | | | |
| Ologen™, Aeon Astron Corporation / iGen™, Life Spring Biotech | Porcine, type I | Skin | **Extraction technology**: Pepsinization  **Processing technology**: Freeze-drying  **Supplements**: Glycosaminoglycans  **Cross-linking:** The agent is not specified | | US7544368B2 | | NCT03506542 [222];  NCT01440751 [225];  NCT02699580 [226]; [227,228] | |
| **(c) Dermatology** | | | | | | | | |
| Integra® Dermal Regeneration Template, Integra LifeSciences | Bovine, type I | Tendon | | **Extraction technology:** Enzymatic and alkali treatment  **Processing technology**: Freeze-drying  **Supplements:** Chondroitin-6-sulfate, silicone layer  **Cross-linking**: Glutaraldehyde | | US6969523B1;  US12059509B1 | | NCT01060670 [250];  NCT03476876 |
| NeoMatriX®, NeXtGen Biologics | Axolotl | Skin | | **Processing technology**: Decellularization, drying/ lyophilization | | US11660376 | | NCT03522675;  NCT03474874 |
| PuraPly™AM, Organogenesis | Porcine | SIS | | **Processing technology**: Decellularization, lamination  **Supplements**: PHMB  **Cross-linking:** EDC | | CA2626460A1 | | NCT03286452 [241];  NCT03070925 [242] |
| Helicoll®, EnColl | Bovine, type I | Skin | | **Extraction technology:** Papain-based treatment, reducing agents  **Processing technology:**  Compressing, dehydrating, dispersing and drying | | US6548077B1 | | NCT06557122 [249] |
| OASIS®, Cook Biotech | Porcine | SIS | | **Processing technology**: Decellularization | | US6206931B1 | | [244–247] |
| PROMOGRAN®, Solventum | Bovine | Skin | | **Processing technology**: Freeze-drying  **Supplements**: Oxidized regenerated cellulose | | US7833790B2 | | [248] |
| **(d) Gastroenterology** | | | | | | | | |
| HEMOPATCH®, Baxter | Bovine | Skin | | **Processing technology**: Freeze-drying  **Supplements**: 4arm-PEG- NHS and 4arm-PEG thiol | | WO2011/079336 | | NCT03410914 [267] |
| Strattice™, LifeCell Corporation | Porcine | Skin | | **Processing technology**: Decellularization, drying | | CA2693613C | | NCT00771407 [269];  NCT02451176 [270] |
| TachoSil®, Corza Medical | Equine | Tendon | | **Processing technology**: foaming, air drying/vacuum drying  **Supplements**: Fibrinogen, thrombin | | WO2002058749A2;  WO2002070594A2;  WO2002058750A2 | | NCT01550406 [31] |
| **(e) Neurosurgery** | | | | | | | | |
| HEMOPATCH®, Baxter | Bovine | Skin | | **Processing technology**: Freeze-drying  **Supplements**: 4arm-PEG- NHS and 4arm-PEG thiol | | WO2011/079336 | | NCT03392662 [278] |
| Lyoplant®, Aesculap AG | Bovine | Pericardium | | **Processing technology**: Decellularization, freeze-drying | | EP0438499B1 | | NCT02678156 [277] |
| **(f) Cardiovascular and general surgery** | | | | | | | | |
| HEMOPATCH®, Baxter | Bovine | Skin | | **Processing technology**: Freeze-drying  **Supplements**: 4arm-PEG- NHS and 4arm-PEG thiol | | WO2011/079336 | | NCT03323359;  NCT04904653;  NCT02633670;  NCT02133378;  NCT04185480 [287] |
| Lyostypt®, Aesculap AG | Bovine, type I | - | | **-** | | - | | NCT00837954 |
| TachoSil®, Corza Medical | Equine | Tendon | | **Processing technology**: Foaming, air drying/vacuum drying  **Supplements**: Fibrinogen, thrombin | | WO2002058749A2;  WO2002070594A2;  WO2002058750A2 | | NCT01192022 [284];  NCT00293514 [285]; NCT05385952 |
| **(g) Orthopedics** | | | | | | | | |
| BEAR® Implant, Miach Orthopaedics | Bovine, type I | Connective tissue | | **Extraction technology:** Pepsinization  **Processing technology:** Freeze-drying  **Supplements:** Autologous whole blood (before implantation) | | EP2073788; US12599700B2 | | NCT02664545 [303]; NCT02292004 [304] |
| INFUSE® (InductOs®), Medtronic | Bovine, type I | Achilles tendon | | **Extraction technology:** Alkali treatment  **Processing technology:** Freeze-drying  **Sterilization:** Ethylene oxide  **Supplements:** rhBMP-2 (before implantation) | | US20070142916A1 | | NCT00853489 [306];  [36,305] |
| HEALOS®, DePuy Synthes | Bovine, type I | - | | **Processing technology:** Freeze-drying  **Supplements:** Hydroxyapatite  **Cross-linking:** The agent is not specified | | - | | [307–309] |
| NOVOCART 3D® | Bovine, type I | Pericardium | | **Processing technology**: Bilayer scaffold formation; unilateral freeze-drying **Supplements:** Autologous chondrocytes (before implantation) | | EP1824420B1 | | [37,310,311] |

**Table S2.** Sponge-form collagen-based medical devices.

| **Product / manufacturer** | **Source and type** | **Tissue / organ** | **Manufacturing technologies** | **Related patents** | **Clinical trials** |
| --- | --- | --- | --- | --- | --- |
| **(a) Dermatology** | | | | | |
| Collost®, BioPHARMAHOLDING | Bovine, type I | Skin | **Processing technology**: Alkali treatment  **Vehicle:** Glucose solution | RU2353397C2 | NCT03677258 |
| Zyplast®, Allergan | Bovine, type I/III | Skin | **Extraction technology**: Pepsinization  **Vehicle:** PBS  **Supplements**: Lidocaine  **Cross-linking**: Glutaraldehyde | US4582640A;  WO1993017075A1 | NCT00876265 |
| Cosmoplast®, Allergan | Human recombinant, type I | Fibroblasts | **Extraction technology**: From cell culture  **Vehicle:** PBS  **Supplements**: Lidocaine  **Cross-linking**: Glutaraldehyde | - | NCT00444210 [324]; NCT00444353 [325] |
| Evolence®, ColBar LifeScience | Porcine, type I | Tendon | **Extraction technology**: Pepsinization  **Vehicle:** PBS  **Cross-linking**: Sugar cross-linking, GLYMATRIX® | WO2001079342A2 | [89] |
| Sunmax FULLSGEN®, Sunmax Biotechnology | Porcine, type I | Skin | **Vehicle:** PBS  **Supplements:** Lidocaine  **Cross-linking**: glutaraldehyde + BDDE + EDC-NHS (triple) | US8889626B2 | NCT03844529 [73,315] |
| BellaFill®, Tiger Aesthetics Medical | Bovine, type I | Skin | **Vehicle:** PBS  **Supplements:** PMMA microspheres, lidocaine | US9789222B2;  US9370469B2; US9370470B2; US9370603B2 | NCT03327311[326];  NCT02642627 [327];  NCT02643628 [328] |
| **(b) Dentistry** | | | | | |
| OsteoBiol® Gel 40, Tecnoss | Porcine, type I/III |  | **Supplements**: 60% bone granular mixture (<300 μm) | - | NCT04782921 [340] |
| OsteoBiol® MP3, Tecnoss Dental | Porcine, type I/III |  | **Supplements**: 90% bone granular mixture (600-1000 or 1000-2000 μm) | - | NCT02644070 [341] |
| **(с) Coloproctology** | | | | | |
| Permacol® collagen paste, Sofradim Production | Porcine, type I | Skin | **Extraction technology**: Decellularization  **Processing technology:** Homogenization  **Vehicle:** NaCl solution  **Cross-linking:** The agent is not specified | - | NCT01624350 [352];  NCT01528995 [354] |
| **(d) Traumatology and orthopedics** | | | | | |
| MD-Tissue®, Guna | Porcine, type I | - | **Processing technology:** Hydrolyzation  **Vehicle:** NaCl solution  **Supplements**: Ascorbic acid, pyridoxine hydrochloride, riboflavin, thiamine hydrochloride | - | NCT05464498;  NCT05486078 |
| MD-Shoulder®, Guna | Porcine, type I | - | **Processing technology:** Hydrolyzation  **Vehicle:** NaCl solution  **Supplements**: Iris extract | - | NCT04492748 [359];  NCT05489484 |
| MD-Knee®, Guna | Porcine, type I | - | **Processing technology:** Hydrolyzation  **Vehicle:** NaCl solution  **Supplements**: Arnica extract | - | [385] |
| RegenSeal®, Cellontech | Porcine, type I | Skin | **Extraction technology**: Pepsinization | US7781158B2 | [371] |
| CartiFill®, Cellontech | Porcine, type I | Skin | **Extraction technology**: Pepsinization  **Vehicle:** PBS | US7781158B2;  KR101863532B1 | NCT02519881 [383];  NCT02539030 [384] |
| CartiZol®, Cellontech | Porcine, type I | Skin | **Extraction technology**: Pepsinization | US7781158B2 | NCT02539095;  NCT07511777;  [386] |
| Fibroquel®, ASPID | Porcine, type I | Skin | **Extraction technology:** Pepsinization  **Vehicle:** Citrate buffer solution  **Supplements:** Polyvinylpyrrolidone  **Cross-linking:** γ-irradiation | EP1752162B1 | NCT04019782 |
| BioCartilage®, Arthrex | Human allogeneic, type II | Cartilage | **Processing technology:** Dehydration and micronization | US10729549B2 | NCT02203071; NCT03696394 |

**Table S3.** Injectable-form collagen-based medical devices.

**Abbreviations: SIS**- small intestinal submucosa, **SMRT™** - Scaffold Matrix Regenerative Therapy, **PHMB** - polyhexamethylene biguanide, **EDC** - ethyl-3-(3-dimethylaminopropyl) carbodiimide, **4-arm PEG-NHS** – 4-arm polyethylene glycol - N-hydroxysuccinimide, **4-arm PEG thiol** – 4-arm polyethylene glycol thiol, **PBS** - phosphate-buffered saline, **BDDE** - 1,4-butanediol diglycidyl ether, **EDC-NHS** – 1-ethyl-3-(3-dimethylaminopropyl) carbodiimide - N-hydroxysuccinimide, **PMMA** - polymethylmethacrylate, **UV** - ultraviolet, **HEMA** - 2-hydroxyethyl methacrylate, **MHBPH** - 4-methacryloxy-2-hydroxybenzophenone, **rhBMP-2** - recombinant human bone morphogenetic protein-2, **ICL** - implantable collamer lens.
